# Safety, tolerability and antiviral activity of the antisense oligonucleotide bepirovirsen in patients with chronic hepatitis B: a phase 2 randomized controlled trial

**DOI:** 10.1038/s41591-021-01513-4

**Published:** 2021-10-12

**Authors:** Man-Fung Yuen, Jeong Heo, Jeong-Won Jang, Jung-Hwan Yoon, Young-Oh Kweon, Sung-Jae Park, Yvonne Tami, Shihyun You, Phillip Yates, Yu Tao, Jennifer Cremer, Fiona Campbell, Robert Elston, Dickens Theodore, Melanie Paff, C. Frank Bennett, T. Jesse Kwoh

**Affiliations:** 1 Department of Medicine and State Key Laboratory of Liver Research, Queen Mary Hospital, The University of Hong Kong, Hong Kong, China; 2 College of Medicine, Pusan National University and Biomedical Research Institute, Pusan National University Hospital, Busan, Republic of Korea; 3 Seoul St. Mary’s Hospital, The Catholic University of Korea, Seoul, Republic of Korea; 4 Seoul National University Hospital, Seoul, Republic of Korea; 5 Kyungpook National University Hospital, Daegu, Republic of Korea; 6 Inje University Busan Paik Hospital, Busan, Republic of Korea; 7 Ionis Pharmaceuticals Inc, Carlsbad, CA USA; 8 GlaxoSmithKline, Collegeville, PA USA; 9 GlaxoSmithKline, Stevenage, UK; 10 GlaxoSmithKline, Research Triangle Park, NC USA

**Keywords:** Infectious diseases, Diseases

## Abstract

Chronic infection with hepatitis B virus (HBV) leads to an increased risk of death from cirrhosis and hepatocellular carcinoma. Functional cure rates are low with current treatment options (nucleos(t)ide analogs (NAs) and pegylated interferons). Bepirovirsen is an antisense oligonucleotide targeting all HBV messenger RNAs; in cell culture and animal models, bepirovirsen leads to reductions in HBV-derived RNAs, HBV DNA and viral proteins. This phase 2 double-blinded, randomized, placebo-controlled trial is the first evaluation of the safety and activity of an antisense oligonucleotide targeting HBV RNA in both treatment-naïve and virally suppressed individuals with chronic HBV infection. The primary objective was to assess the safety and tolerability of bepirovirsen in individuals with chronic hepatitis B (CHB) (NCT02981602). The secondary objective was to assess antiviral activity, including the change from baseline to day 29 in serum hepatitis B surface antigen (HBsAg) concentration. Participants with CHB infection ≥6 months and serum HBsAg ≥50 IU ml^−1^ were enrolled from seven centers across Hong Kong and the Republic of Korea and randomized (3:1 within each dose cohort) to receive bepirovirsen or placebo via subcutaneous injection twice weekly during weeks 1 and 2 (days 1, 4, 8 and 11) and once weekly during weeks 3 and 4 (days 15 and 22). Participants were then followed for 26 weeks. Twenty-four participants were treatment-naïve and seven were receiving stable NA therapy. Treatment-emergent adverse events were mostly mild/moderate (most commonly injection site reactions). Eleven (61.1%) and three (50.0%) treatment-naïve participants experienced one or more treatment-emergent adverse event in the bepirovirsen and placebo groups, respectively. In participants receiving NA therapy, the corresponding numbers were three (60.0%) and one (50.0%). Transient, self-resolving alanine aminotransferase flares (≥2× upper limit of normal) were observed in eight treatment-naïve participants and three participants on stable NA regimens in the bepirovirsen treatment arms. HBsAg reductions were observed and were significant versus placebo for treatment-naïve participants receiving bepirovirsen 300 mg (*P* = 0.001), but not for the bepirovirsen 150 mg group (*P* = 0.245) or participants receiving stable NA therapy (*P* = 0.762). Two participants in each of the 300 mg dose groups achieved HBsAg levels below the lower limit of quantitation by day 29 (*n* = 3) or day 36 (*n* = 1). Bepirovirsen had a favorable safety profile. These preliminary observations warrant further investigation of the safety and activity of bepirovirsen in a larger CHB patient population.

## Abstract (web summary)

A first-in-human study of an antisense oligonucleotide targeting hepatitis B virus (HBV) RNA provides initial insights into this potential new therapeutic modality for individuals with chronic HBV infection.

## Main

HBV infection can be chronic, especially if it occurs before 5 years of age, and chronicity results in an increased risk of death from cirrhosis and hepatocellular carcinoma in the long term^1^. In 2019, it was estimated that 296 million people were living with chronic HBV infection, resulting in ~820,000 deaths per year globally in 2019, mostly from cirrhosis and hepatocellular carcinoma (https://www.who.int/news-room/fact-sheets/detail/hepatitis-b)^2^. The World Health Organization’s global target is to reduce new viral hepatitis infections by 90% by 2030 (https://www.who.int/news-room/fact-sheets/detail/hepatitis-b). The treatment goal for patients with CHB is to obtain functional cure, namely, sustained loss of detectable serum HBsAg with undetectable HBV DNA^3^. Functional cure is associated with improved clinical outcomes and allows patients to be off-therapy with a minimal risk of relapse^4–6^. HBsAg plays a major role in maintaining chronic HBV infection by impairing the immune response against HBV^7,8^. Reducing HBsAg expression may permit reconstitution of an immune response against HBV^7^.

First-line therapy for CHB is NAs^1,9^. Current NAs suppress serum HBV DNA levels and improve hepatic fibrosis and cirrhosis^1,4^, but have minimal effect on HBsAg levels^10,11^. Even with life-long therapy, HBsAg seroclearance is uncommon^9^ and there is a high relapse rate after NA withdrawal due to the persistence in hepatocytes of covalently closed circular HBV DNA, which is the transcription template enabling the resumption of HBV DNA replication after NA treatment is ended^10,12^. Pegylated interferons are also approved for CHB treatment for up to 48 weeks^5,9^. However, the tolerability profile of approved pegylated interferons results in many patients being ineligible or unwilling to receive treatment^9^. Treatment-induced functional cure rates are low with current treatment options^9^.

There is a significant unmet need for new CHB therapies that achieve functional cure when administered for a finite duration, enabling patients to control their infection, freeing them from life-long therapy and reducing their risk of hepatocellular carcinoma. Bepirovirsen (previously ISIS 505358; GSK3228836) is an antisense oligonucleotide (ASO) with a 2′-*O*-methoxyethyl (2′-MOE) gapmer design (full sequence is publicly available)^13^. The first five and final five nucleotides are of MOE-modified ribonucleotides; the central ten nucleotides are oligodeoxynucleotides. The drug is uniformly modified with phosphorothioate linkages. The bepirovirsen binding site (GCACTTCGCTTCACCTCTGC) is present in all HBV mRNA and pregenomic RNA; as such, bepirovirsen would be expected to reduce levels of all HBV mRNAs including pregenomic RNA. Specific ASOs bind to complementary HBV RNA transcripts forming a hybrid ASO/RNA complex, which recruits endogenous RNase H, cleaving the HBV RNA and leading to degradation of the transcript. This results in a reduction in HBV-derived RNAs, HBV DNA and viral proteins (including HBsAg) in cell culture and in animal models^14^.

This study examined the safety, tolerability and antiviral activity of multiple doses of bepirovirsen over 4 weeks in patients with CHB.

## Results

### Study participants and dosing

The ISIS 505358-CS3 study (GSK study 205695; ClinicalTrials.gov: NCT02981602) was a phase 2, double-blinded, placebo-controlled, dose-escalation trial of bepirovirsen in 31 patients with CHB who were either treatment-naïve (cohorts 1–3, *n* = 24) or receiving stable NA therapy (cohort 4 (on-NA), *n* = 7) (Supplementary Fig. 1). The study protocol can be accessed at https://www.gsk-studyregister.com/en/trial-details/?id=205695. Treatment-naïve patients were randomized to placebo (*n* = 6), bepirovirsen 150 mg (*n* = 6) or 300 mg (*n* = 12) and on-NA patients to placebo (*n* = 2) or bepirovirsen 300 mg (*n* = 5) (Supplementary Fig. 2). Six doses of bepirovirsen or placebo were administered via subcutaneous injection during the 4-week treatment period: twice weekly during weeks 1 and 2 (days 1, 4, 8 and 11) and once weekly during weeks 3 and 4 (days 15 and 22). Patients were then followed for 26 weeks.

One on-NA patient withdrew from the study on day 8, after two doses of bepirovirsen 300 mg, due to transient mild fevers after each dose that were considered treatment-related; all other patients completed the treatment and follow-up periods. Demographics and baseline characteristics were similar between treatment arms (Table 1).

**Table 1:** Demographics and baseline characteristics in patients with CHB (safety population)

	Treatment-naïve			On-NA	
	Bepirovirsen 150 mg (*n* = 6)	Bepirovirsen 300 mg (*n* = 12)	Placebo (*n* = 6)	Bepirovirsen 300 mg (*n* = 5)	Placebo (*n* = 2)
Age (years)					
Mean (s.d.)	42.5 (11.22)	42.6 (14.12)	49.3 (12.66)	48.4 (7.40)	37.0 (2.83)
Range	23, 52	18, 61	34, 69	40, 59	35, 39
Sex, *n* (%)					
Male	3 (50.0)	4 (33.3)	4 (66.7)	4 (80.0)	1 (50.0)
Female	3 (50.0)	8 (66.7)	2 (33.3)	1 (20.0)	1 (50.0)
Height (cm), mean (s.d.)	166.12 (4.673)	165.97 (8.796)	163.52 (4.436)	166.60 (10.188)	166.50 (6.364)
Weight (kg), mean (s.d.)	66.00 (13.838)	62.25 (9.321)	60.00 (8.199)	66.68 (16.066)	68.60 (4.525)
Race, *n* (%)					
Asian	6 (100.0)	12 (100.0)	6 (100.0)	5 (100.0)	2 (100.0)
HBsAg (log_10_ IU ml^−1^)					
Mean (s.d.)	3.57 (1.244)	3.89 (1.056)	3.21 (1.304)	2.78 (0.363)	3.77 (1.018)
Median	3.85	4.34	3.08	2.80	3.77
Min, max	1.75, 4.78	2.03, 5.04	1.82, 5.25	2.24, 3.20	3.05, 4.49
HBeAg status, *n* (%)					
Positive	5 (83.3)	6 (50.0)	2 (33.3)	0 (0)	0 (0)
Negative	1 (16.7)	6 (50.0)	4 (67.7)	5 (100.0)	2 (100.0)
HBV DNA (log_10_ IU ml^−1^)					
Mean (s.d.)	7.41 (1.396)	6.77 (1.892)	5.57 (2.121)	<1.30 (0.000)^a^	<1.30 (0.000)^a^
Median	7.29	7.20	5.14	<1.30	<1.30
Min, max	5.98, 9.11	3.93, 8.74	3.67, 9.29	NA	NA
HBsAg genotype, *n* (%)					
Type B	2 (33.3)	4 (33.3)	2 (33.3)	0	0
Type C	4 (66.7)	8 (66.7)	4 (66.7)	0	0
Indeterminate	0	0	0	5 (100.0)^b^	2 (100.0)^b^
Anti-HBs antibody presence, *n* (%)	1 (16.7)	0	1 (16.7)	0	0
Anti-HBe antibody presence, *n* (%)	1 (16.7)	6 (50.0)	4 (66.7)	4 (80.0)	2 (100.0)
ALT (U l^−1^)					
Mean (s.d.)	53.2 (35.52)	42.9 (31.77)	35.8 (13.29)	20.8 (9.31)	15.0 (7.07)
Min, max	24, 107	11, 103	22, 51	12, 34	10, 20
AST (U l^−1^)					
Mean (s.d.)	35.5 (19.56)	33.3 (16.42)	29.8 (13.06)	21.4 (5.64)	17.0 (8.49)
Min, max	17, 64	15, 65	18, 55	16, 28	11, 23

^a^Because all participants in cohort 4 were stably maintained on NA, they had undetectable HBV DNA levels at baseline. This table presents log_10_ values of either <20 IU ml^−1^ or ‘target not detected’.

^b^Historical data not available and HBV DNA levels were below the LLOQ to establish genotyping.

Anti-HBe antibody, antibody to HBV e antigen; anti-HBs, antibody to HBV surface antigen.

### Safety and tolerability

The most common treatment-emergent adverse events (TEAEs) were local injection site reactions (Table 2). Injection site reactions were reported in zero, three (50%) and three (25%) treatment-naïve patients in the placebo, bepirovirsen 150 mg and bepirovirsen 300 mg arms, respectively, and in zero and two (40%) on-NA patients in the placebo and bepirovirsen 300 mg arms, respectively. Pyrexia was commonly reported in treatment-naïve patients (one patient (16.7%) each in the placebo and bepirovirsen 150 mg arms; three patients (25%) in the bepirovirsen 300 mg arm).

**Table 2:** Summary of TEAEs by system organ class and preferred term in patients with CHB (safety population)

TEAE, *n* (%) system organ class preferred term	Treatment-naïve				On-NA	
	Bepirovirsen 150 mg (*n* = 6)	Bepirovirsen 300 mg (*n* = 12)	Total (*n* = 18)	Placebo (*n* = 6)	Bepirovirsen 300 mg (*n* = 5)	Placebo (*n* = 2)
Patients with ≥1 TEAE	5 (83.3)	6 (50.0)	11 (61.1)	3 (50.0)	3 (60.0)	1 (50.0)
General disorders and administration site conditions	4 (66.7)	5 (41.7)	9 (50.0)	2 (33.3)	3 (60.0)	0
Chest discomfort	0	1 (8.3)	1 (5.6)	0	0	0
Influenza-like illness	0	0	0	1 (16.7)	0	0
Injection site bruising	1 (16.7)	0	1 (5.6)	0	1 (20.0)	0
Injection site erythema	0	3 (25.0)	3 (16.7)	0	1 (20.0)	0
Injection site pain	1 (16.7)	1 (8.3)	2 (11.1)	0	0	0
Injection site pruritus	2 (33.3)	1 (8.3)	3 (16.7)	0	0	0
Injection site rash	1 (16.7)	0	1 (5.6)	0	0	0
Injection site swelling	0	2 (16.7)	2 (11.1)	0	1 (20.0)	0
Pyrexia	1 (16.7)	3 (25.0)	4 (22.2)	1 (16.7)	1 (20.0)	0
Gastrointestinal disorders	2 (33.3)	3 (25.0)	5 (27.8)	1 (16.7)	0	0
Abdominal discomfort	1 (16.7)	0	1 (5.6)	0	0	0
Abdominal pain upper	0	1 (8.3)	1 (5.6)	0	0	0
Gastritis	0	0	0	1 (16.7)	0	0
Mouth swelling	1 (16.7)	0	1 (5.6)	0	0	0
Nausea	1 (16.7)	2 (16.7)	3 (16.7)	1 (16.7)	0	0
Investigations	0	3 (25.0)	3 (16.7)	0	0	0
ALT increased	0	2 (16.7)	2 (11.1)	0	0	0
CRP increased	0	1 (8.3)	1 (5.6)	0	0	0
Blood and lymphatic system disorders	1 (16.7)	1 (8.3)	2 (11.1)	0	0	0
Anemia	1 (16.7)	1 (8.3)	2 (11.1)	0	0	0
Infections and infestations	0	2 (16.7)	2 (11.1)	0	0	0
Hand-foot-and-mouth disease	0	1 (8.3)	1 (5.6)	0	0	0
Influenza	0	1 (8.3)	1 (5.6)	0	0	0
Musculoskeletal and connective tissue disorders	0	2 (16.7)	2 (11.1)	0	0	0
Myalgia	0	2 (16.7)	2 (11.1)	0	0	0
Nervous system disorders	0	2 (16.7)	2 (11.1)	1 (16.7)	0	1 (50.0)
Headache	0	2 (16.7)	2 (11.1)	1 (16.7)	0	1 (50.0)
Skin and subcutaneous tissue disorders	0	2 (16.7)	2 (11.1)	1 (16.7)	0	0
Post inflammatory pigmentation change	0	1 (8.3)	1 (5.6)	0	0	0
Pruritus generalized	0	1 (8.3)	1 (5.6)	0	0	0
Rash maculo-papular	0	0	0	1 (16.7)	0	0
Urticaria	0	1 (8.3)	1 (5.6)	0	0	0
Injury, poisoning, and procedural complications	0	1 (8.3)	1 (5.6)	0	0	0
Contusion	0	1 (8.3)	1 (5.6)	0	0	0

TEAEs were mostly mild (division of acquired immune deficiency syndrome (DAIDS) grade 1: 52 of 69 events); the remainder were moderate (DAIDS grade 2: 16 of 69 events), except for one treatment-naïve patient in the bepirovirsen 300 mg treatment group who experienced a serious adverse event (DAIDS grade 4) of alanine aminotransferase (ALT) increase (described in the ‘ALT increase’ section).

Treatment-related TEAEs were reported in four (66.7%), six (50.0%) and one (16.7%) treatment-naïve patients in the bepirovirsen 150 mg, bepirovirsen 300 mg and placebo groups, respectively. The most common treatment-related TEAEs in treatment-naïve patients were injection site pruritus (bepirovirsen 150 mg, *n* = 2 (33%); bepirovirsen 300 mg, *n* = 1 (8.3%)), injection site erythema (bepirovirsen 150 mg, *n* = 0 (0%); bepirovirsen 300 mg, *n* = 3 (25%)) and nausea (bepirovirsen 150 mg, *n* = 1 (16.7%); bepirovirsen 300 mg, *n* = 2 (16.7%)). Injection site swelling, ALT increase and myalgia were each considered treatment-related in two patients (16.7%) in the bepirovirsen 300 mg arm. In on-NA patients, injection site bruising, injection site swelling, injection site erythema and pyrexia were each reported in one patient (20.0%) in the bepirovirsen 300 mg arm.

An increase in C-reactive protein (CRP) levels following the first dose of bepirovirsen was observed in patients with CHB. In most patients, levels increased on day 2 with peak levels observed pre-dose on day 4 (see example patient in Supplementary Fig. 3a). Levels were substantially recovered by day 8, suggesting the day 4 dose did not lead to further CRP increases. There were generally no CRP spikes at later time points, and no symptoms were consistently associated with CRP elevations. CRP elevations were dose-related in patients and were consistent with those observed in healthy volunteers (Supplementary Fig. 3b and unpublished data).

Aside from ALT, aspartate aminotransferase (AST; described below) and CRP effects, there were no clinically significant changes in laboratory tests related to bepirovirsen treatment. Transient prolongations in activated partial thromboplastin time were observed 3–5 h after administration of both bepirovirsen doses on day 1 and day 22. The magnitudes of these prolongations were not clinically relevant, with a maximum observed value of 43.9 s (~1.21× upper limit of normal (ULN); ULN = 36.5 s) in two participants in the bepirovirsen 300 mg treatment group. There were no bleeding or bruising events associated with the elevations and little or no coincidental prolongation of prothrombin time.

There were no observations of complement activation related to bepirovirsen dosing and no clinically significant findings in other safety assessments (for example, electrocardiogram, physical examination, concomitant medication usage).

### Efficacy

#### Change from baseline in HBsAg

A dose-dependent reduction from baseline in HBsAg was observed at day 29 (7 d after the last dose) in the bepirovirsen treatment arms. For treatment-naïve patients, the mean (s.d.) HBsAg reduction from baseline to day 29 was 0.50 (0.57) log_10_ IU ml^−1^ (*P* = 0.245 versus placebo) and 1.56 (1.38) log_10_ IU ml^−1^ (*P* = 0.001 versus placebo) in the bepirovirsen 150 mg and 300 mg arms, respectively (Table 3). For on-NA patients, the mean (s.d.) HBsAg reduction to day 29 was 1.99 (1.80) log_10_ IU ml^−1^ (Table 3). By contrast, no placebo-treated patient had a reduction in HBsAg of >0.07 log_10_ IU ml^−1^ by day 29. Four patients achieved transient HBsAg loss, defined as HBsAg level below the lower limit of quantitation (LLOQ: 0.05 IU ml^−1^), by day 29 (*n* = 3) or by day 36 (*n* = 1).

**Table 3:** HBsAg and HBV DNA at baseline and day 29 in patients with CHB (full analysis population)

	Treatment-naïve			On-NA	
	Bepirovirsen 150 mg (*n* = 6)	Bepirovirsen 300 mg (*n* = 12)	Placebo (*n* = 6)	Bepirovirsen 300 mg (*n* = 5)	Placebo (*n* = 2)
Baseline HBsAg (log_10_ IU ml^−1^)					
*n*	6	12	6	5	2
Mean (s.d.)	3.57 (1.244)	3.89 (1.056)	3.21 (1.304)	2.78 (0.363)	3.77 (1.018)
Day 29 HBsAg (log_10_ IU ml^−1^), LOCF					
*n*	6	12	6	5	2
Mean (s.d.)	3.06 (1.580)	2.34 (2.232)	3.21 (1.237)	0.79 (2.118)	3.76 (0.979)
Change from baseline to day 29 in HBsAg (log_10_ IU ml^−1^), LOCF					
*n*	6	12	6	5	2
Mean (s.d.)	–0.50 (0.566)	–1.56 (1.379)	0.00 (0.112)	–1.99 (1.799)	–0.01 (0.039)
*P* value (versus placebo)	0.245	0.001		0.762	
Baseline HBV DNA (log_10_ IU ml^−1^)					
*n*	6	12	6	5	2
Mean (s.d.)	7.41 (1.396)	6.77 (1.892)	5.57 (2.121)	<1.30 (0.000)	<1.30 (0.000)
Day 29 HBV DNA (log_10_ IU ml^−1^), LOCF					
*n*	6	12	6	5	2
Mean (s.d.)	7.03 (1.451)	5.12 (3.073)	5.57 (2.429)	1.37 (0.167)	<1.30 (0.000)
Change from baseline to day 29 in HBV DNA (log_10_ IU ml^−1^), LOCF					
*n*	6	12	6	5	2
Mean (s.d.)	–0.38 (0.420)	–1.66 (1.479)	0.00 (0.471)	0.08 (0.167	0.00 (0.000)
*P* value (versus placebo)	0.116	<0.001		NA	

Baseline was the last nonmissing measurement before the first dose of the study drug. The last observation carried forward (LOCF) method was used to impute missing values. Comparison between bepirovirsen and pooled placebo was performed for each dose level separately using an analysis of covariance model with baseline as a covariate and treatment group as a factor. All comparisons were prespecified with no adjustment for multiple comparisons. Two-sided *P* values are presented.

**Fig. 1:**
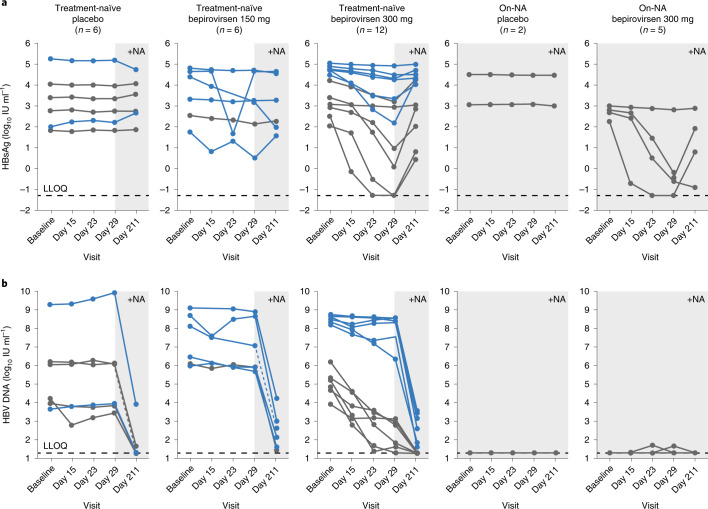
Change over time in serum HBsAg and HBV DNA. **a**,**b**, HBsAg (**a**) and HBV DNA (**b**) at baseline, day 15, day 23, day 29 and end of study (day 211) in patients with CHB (full analysis population). Blue lines indicate HBeAg-positive patients; gray lines indicate HBeAg-negative patients; black horizontal dashed line indicates LLOQ; gray shading indicates NA administration. In HBV DNA panels for treatment-naïve patients treated with placebo and bepirovirsen 150 mg, dashed lines between day 29 and day 211 are for patients whose day 211 test failed to produce a result. The last observation for these two patients (day 113) was carried forward to day 211. Four patients reached undetectable HBsAg levels; however, only three of the four reached LLOQ by day 29, the fourth patient reached LLOQ by day 36. Comparison between bepirovirsen and pooled placebo was performed for each dose level, separately, using an analysis of covariance model with baseline as a covariate and treatment group as a factor. For each comparison, if data departed substantially from normality, the Wilcoxon rank sum test was used.

**Fig. 2:**
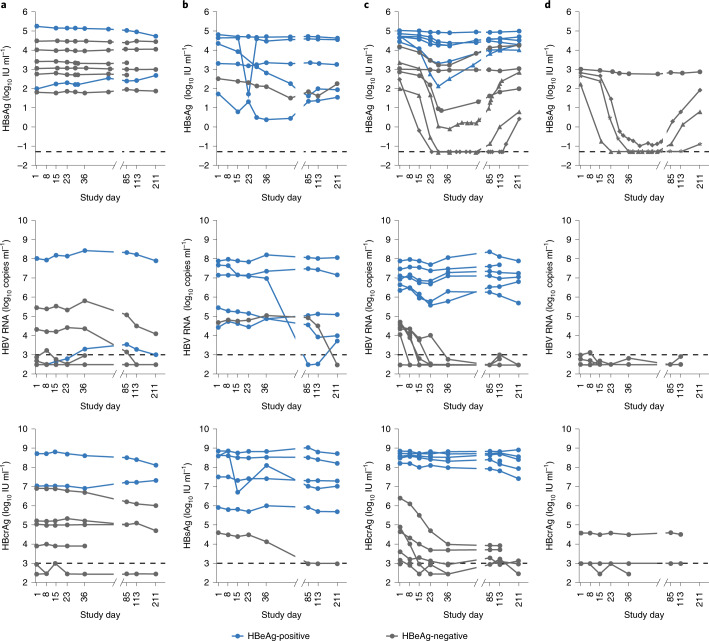
Change in HBsAg, HBV RNA and HBcrAg (all time points). **a**, Placebo (NA-naïve and NA-treated; *n* = 8). **b**, Bepirovirsen 150 mg (NA-naïve; *n* = 6). **c**, Bepirovirsen 300 mg (NA-naïve; *n* = 12). **d**, Bepirovirsen 300 mg (NA-treated; *n* = 4*) (full analysis population; HBV RNA and HBcrAg analyses were post hoc). One patient discontinued treatment on study day 8 and is not shown. This patient discontinued treatment and withdrew from the study; as such they were not assessed beyond day 8 and are not shown in this figure (exclusion not prespecified), this patient was also excluded from the per-protocol population (according to prespecified criteria). Dotted lines denote LLOQ. The data shown are descriptive, no statistical analysis was conducted.

Six of 12 treatment-naïve patients receiving bepirovirsen 300 mg had HBsAg reduction ≥1.0 log_10_ IU ml^−1^ at day 29 (an additional patient had 0.98 log_10_-transformed reduction) (Fig. 1a). Three of these six patients experienced a reduction of ≥3.0 log_10_ and two achieved HBsAg loss. HBsAg loss was transient in one patient (Fig. 2 and Supplementary Fig. 4c), from day 29 to day 57, with measurable levels (0.14 IU ml^−1^) detected on day 85, but was more prolonged in the second patient (from day 23 maintained to day 126, with measurable levels on day 140 (0.14 IU ml^−1^)) (Fig. 2 and Supplementary Fig. 4).

Three of four on-NA patients completing bepirovirsen 300 mg dosing had a HBsAg reduction ≥3.0 log_10_ at day 29; of these three patients, one reached HBsAg levels below LLOQ on day 29 and another on day 36, whereas the third patient did not reach HBsAg levels below LLOQ. Similar to the treatment-naïve patients, one patient had transient HBsAg loss (Fig. 2), from day 23 to day 57, with measurable levels detected on day 85 (0.14 IU ml^−1^), but a second patient experienced a more prolonged HBsAg loss from day 36 to day 113, inclusive, with measurable levels detected on day 211 (0.12 IU ml^−1^; maintaining a 3.59 log_10_ IU ml^−1^ lower HBsAg level than baseline by the end of the study; Fig. 2 and Supplementary Fig. 4). The fourth patient had no decrease in HBsAg. Individual HBsAg levels over time by baseline HBsAg concentration are shown in Supplementary Fig. 5.

Reductions in HBsAg were detected in both hepatitis B e-antigen (HBeAg)-positive and HBeAg-negative patients, providing confidence that the target sequence for bepirovirsen is present even when HBsAg may be derived from integrated genomes^15^. Baseline HBsAg levels were generally lower in HBeAg-negative patients than in HBeAg-positive patients, and the log_10_-transformed reduction in HBsAg from baseline to day 29 was generally of a greater magnitude in HBeAg-negative patients compared with HBeAg-positive patients treated with bepirovirsen 300 mg (Fig. 1a). All four patients who reached undetectable HBsAg levels were HBeAg negative.

#### Change from baseline in HBV DNA

A dose-dependent reduction from baseline in HBV DNA was observed at day 29 (7 d after the last dose and before initiation of NA therapy) in treatment-naïve patients who received bepirovirsen. The mean (s.d.) HBV DNA reduction from baseline to day 29 was 0.38 (0.42) log_10_ IU ml^−1^ (*P* = 0.116 versus placebo) and 1.66 (1.48) log_10_ IU ml^−1^ (*P* < 0.001 versus placebo) in the bepirovirsen 150 mg and 300 mg arms, respectively (Table 3). After day 29, HBV DNA levels declined substantially in all patients, as expected with the administration of NA treatment (Fig. 1b).

All six treatment-naïve patients who received bepirovirsen 300 mg and had a >1.0 log_10_ IU ml^−1^ reduction in plasma HBsAg also had a >0.5 log_10_ IU ml^−1^ reduction in HBV DNA by day 29; with the three patients who experienced a ≥3 log_10_ IU ml^−1^ reduction in HBsAg also having a >3 log_10_ IU ml^−1^ reduction in HBV DNA by day 29 (Supplementary Fig. 6). One of the patients with a >3.0 log_10_ IU ml^−1^ reduction in HBV DNA had levels below the LLOQ (20 IU ml^−1^) by day 29, before initiation of tenofovir treatment. A fourth patient, who had a 0.98 log_10_ IU ml^−1^ reduction in plasma HBsAg at day 29, also had a >3 log_10_ IU ml^−1^ reduction in HBV DNA.

Although reductions in HBV DNA were observed in both HBeAg-positive and HBeAg-negative patients at day 29, the reduction was generally greater in HBeAg-negative patients. Baseline HBV DNA was substantially higher for HBeAg-positive patients, and fewer patients with higher DNA levels at day 29 had a reduction to the LLOQ at day 211 (Fig. 1b).

### ALT increases

ALT flares, defined as an ALT level of ≥2× ULN, were observed during this study. Of the 18 treatment-naïve patients administered bepirovirsen, eight experienced an ALT flare: two patients in the bepirovirsen 150 mg arm and six in the bepirovirsen 300 mg arm. Among these patients, both those in the 150 mg arm and three of six patients in the 300 mg arm had an ALT level ≥2× ULN at baseline, which further increased during treatment. In the on-NA group, all patients had a normal ALT level at baseline; however, three of five patients receiving bepirovirsen 300 mg experienced an ALT flare. All ALT flares were transient and self-resolved. AST increases from baseline of at least two grades were observed in two treatment-naïve patients and two on-NA patients following treatment with bepirovirsen; these changes were concurrent with ALT increases but were of a smaller magnitude. There were no concurrent changes in bilirubin (total and direct) for any patient.

**Fig. 3:**
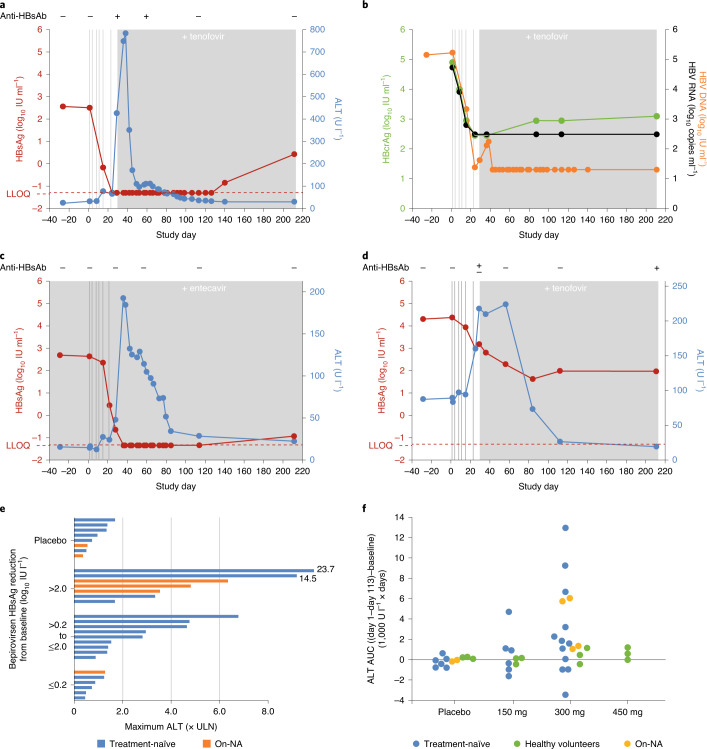
Profiles and relationships of ALT, HBsAg, HBcrAg and HBV DNA levels in treatment-naïve, NA-treated patients and healthy volunteers receiving different doses of bepirovirsen. **a**,**b**, HBsAg and ALT (**a**), and HBcrAg, HBV RNA and HBV DNA (**b**) in a treatment-naïve patient treated with bepirovirsen 300 mg. **c**, HBsAg and ALT in a patient already on entecavir treated with bepirovirsen 300 mg. **d**, HBsAg and ALT in a treatment-naïve patient treated with bepirovirsen 150 mg (vertical lines indicate dose administration days; gray shading indicates NA dosing period for treatment-naïve patients). **e**, ALT results at day 29 categorized by HBsAg reduction from baseline. **f**, ALT AUC categorized by dose group in patients with CHB (safety population) and healthy volunteers (Study CS1 safety population; unpublished data) receiving bepirovirsen (*y* axis is the AUC of ALT from day 1 to day 113 that is above the AUC of baseline ALT maintained for the time period). +, positive for anti-HBsAb; –, negative for anti-HBsAb; ±, indeterminate anti-HBsAb status; anti-HBsAg, antibody to HBV surface antigen. Data shown are descriptive, no statistical analysis was conducted.

Reductions in HBsAg either preceded the ALT increases or occurred concomitantly with ALT flares (Fig. 3a–d and Supplementary Fig. 4). Generally, larger ALT increases (based on peak ALT) were associated with greater HBsAg reductions (Fig. 3e and Supplementary Fig. 6). For patients with a HBsAg reduction ≥3.0 log_10_, ALT flares were observed for all on-NA patients (three) and for two of three treatment-naïve patients. In treatment-naïve patients, larger ALT increases were also associated with greater HBV DNA reductions at day 29 (Supplementary Fig. 7). By contrast, no ALT flares were observed in patients without a HBsAg reduction (<0.2 log_10_) or in patients receiving placebo. Comparison of ALT area under the curve (AUC) for days 1–113 by bepirovirsen dose level in patients with CHB and in healthy volunteers (unpublished data) when dosed for 4 weeks, shows ALT increases in the latter population were minor even at a higher dose than observed in patients with CHB (Fig. 3f).

The highest ALT concentration observed was 781 U l^−1^ (23.7× ULN; ~26-fold change from baseline), which was recorded as a serious adverse event 15 d after the last dose of bepirovirsen 300 mg (week 6) in a treatment-naïve patient (Fig. 3a and Supplementary Fig. 8a). Increased AST was also observed in this patient (up to 525 U l^−1^ at the same time point; 21-fold change from baseline). ALT and AST levels decreased substantially within 2 weeks and returned to baseline by week 17. The patient was asymptomatic throughout. Alkaline phosphatase elevation (1.7× ULN) was also observed in this patient (Supplementary Fig. 8a); all other patients with ALT increases had no notable change in alkaline phosphatase. Another treatment-naïve patient in the bepirovirsen 300 mg arm had an ALT flare ≥10× ULN, reaching a maximum ALT increase of 479 U l^−1^ at week 6 (approximately sevenfold change from baseline) (Supplementary Figs. 4d and 8b). This patient also had an associated increase in AST up to 343 U l^−1^ at week 6 (approximately sevenfold change from baseline). In the on-NA cohort, three patients experienced ALT flares, which were temporally associated with a reduction in HBsAg. These ALT increases were <10× ULN, with maximum ALT levels <300 IU l^−1^.

### Change from baseline in the virological biomarkers HBV RNA, HBcrAg and HBeAg

Of the six treatment-naïve patients receiving bepirovirsen 300 mg and having a ≥1.0 log_10_-transformed reduction in HBsAg (IU ml^−1^) at day 29, five and four, respectively, also had a concomitant >0.5 log_10_-transformed reduction by day 36 in HBV RNA (copies ml^−1^) and hepatitis B core-related antigen (HBcrAg; IU ml^−1^) (Supplementary Fig. 6). By contrast, both treatment-naïve patients receiving bepirovirsen 300 mg and having ≤0.2 log_10_-transformed reduction in HBsAg, also had minimal change (≤0.2 log_10_-transformed reduction) in HBV RNA and HBcrAg (Supplementary Fig. 6). Analysis was not possible in the on-NA patients because HBV RNA and HBcrAg at baseline were close to or below the LLOQ (as expected for patients receiving NA therapy). HBV RNA and HBcrAg responses over time are shown in Fig. 2.

In the 13 patients who were HBeAg-positive at baseline, minimal reductions in serum HBeAg concentration (IU ml^−1^) were observed from baseline to day 29 and to week 31 (Supplementary Table 1). Reductions in HBeAg of the magnitude observed for HBsAg and HBV DNA were generally not observed, other than for the patient shown in Fig. 3d. In this patient, HBeAg was 780 IU ml^−1^ at baseline and reduced by 1.0 log_10_ on day 22, 0.5 log_10_ on day 29, 0.9 log_10_ on day 36, 3.5 log_10_ on day 113 and 3.0 log_10_ on day 211 (Supplementary Fig. 9).

### Seroconversion to anti-HBs or anti-HBe antibodies

Seroconversion was assessed as an exploratory objective. Transient treatment-emergent positivity for anti-HBs antibody, based on a qualitative test performed with the cut-off of ≥11.5 mIU ml^−1^, was observed for one of six and five of twelve treatment-naïve patients who received bepirovirsen 150 mg and 300 mg, respectively, and for one of five on-NA patients who received bepirovirsen 300 mg. The treatment-naïve patient treated with 150 mg was positive on day 22 and indeterminate on day 29 (Fig. 3d). The six patients treated with bepirovirsen 300 mg were all positive on day 29; four treatment-naïve patients and one on-NA patient were positive and indeterminate, respectively, on day 57 (for examples, see Fig. 3a and Supplementary Fig. 4a,d). Two treatment-naïve patients and one on-NA patient with ≥3.0 log_10_ IU ml^−1^ HBsAg reduction at day 29 experienced transient anti-HBs antibody positivity.

No patients who were anti-HBe negative at baseline, in either group, had anti-HBe antibody positivity at day 29. The treatment-naïve patient shown in Fig. 3d, who had a substantial and persistent reduction in HBsAg, was negative for anti-HBe antibody from screening to day 29 but was positive from day 57 to the end of study (day 211), which coincides with the period when their HBeAg levels were ≤0.6 log_10_ below baseline level. One patient in the on-NA group had detectable HBeAg at screening, day 1 and day 15, although they were considered HBeAg negative because all values were <0.09 IU ml^−1^. From day 23, the patient’s HBeAg levels were persistently below the LLOQ (<0.06 IU ml^−1^); however, on day 211 (final study visit) this patient tested positive for anti-HBe antibody.

### Bepirovirsen binding site sequencing

The sequence of the bepirovirsen binding site was assessed via DNA or RNA sequencing (Supplementary Results and Supplementary Fig. 10). No sequence alterations were found in any of the samples assessed at baseline (*n* = 25), day 29 (*n* = 17) and day 113 (*n* = 7).

## Discussion

This is the first study in which bepirovirsen has been administered to patients with CHB. A dose-dependent reduction in HBsAg and HBV DNA after 4 weeks of treatment with bepirovirsen (150 or 300 mg) was observed in treatment-naïve patients with CHB, with a statistically significant reduction in HBsAg and HBV DNA compared with placebo in the bepirovirsen 300 mg treatment group, but not in the bepirovirsen 150 mg group. Reductions in HBsAg were also seen with bepirovirsen 300 mg treatment in patients on stable NA therapy but were not statistically significant. However, the absolute mean change from baseline in patients treated with bepirovirsen 300 mg was higher in the NA-treated group than in the treatment-naïve group, indicating that the lack of statistical significance may be due to the small sample size. Among the most robust responders, the HBsAg response was rapid and was prolonged after the end of treatment; however, the durability of response was variable. Given that HBsAg loss is a rare event with currently available treatments^9^, the possibility of a rapid and prolonged HBsAg response after only 4 weeks of bepirovirsen treatment supports further exploration of this dosing regimen and treatment duration in a larger number of patients to determine whether functional cure is possible. Additionally, the greatest HBsAg log declines were observed in patients with low baseline HBsAg levels, highlighting the importance of baseline HBsAg levels on HBsAg decline (at low baseline HBsAg values, an absolute HBsAg decrease was equivalent to a greater log decline).

HBeAg-negative patients represent the majority of those with CHB, and its prevalence has increased over the past decade^1,16^. HBeAg-negative patients tend to have lower HBV DNA levels and be in a later stage of infection than those who are HBeAg positive^9^. HBsAg reduction was observed in both HBeAg-negative and HBeAg-positive patients treated with bepirovirsen 300 mg; however, greater log_10_-transformed reductions in HBsAg (and HBV DNA) were observed in HBeAg-negative patients. Wooddell et al. have previously demonstrated that a substantial proportion of HBsAg in HBeAg-negative patients is derived from integrated HBV sequences and integration can lead to the loss of target sequences in the HBV mRNA^15^. The substantial reduction of HBsAg in HBeAg-negative patients suggests that the bepirovirsen target site is preserved in the majority of transcripts derived from integrated HBV genomes.

Compared with HBeAg-positive patients, HBeAg-negative patients had lower baseline HBsAg and HBV DNA, which may be a factor responsible for the improved response. These findings suggest that high baseline antigen and viral loads may be factors contributing negatively to the response. The observation that a higher viral load at day 29 appeared to be a negative factor for achieving a reduction in HBV DNA with NA treatment to below LLOQ at day 211 supports this possibility. Most HBeAg-positive patients also showed a minimal reduction in HBeAg after treatment, except for one patient in the bepirovirsen 150 mg group, who had a substantial HBeAg reduction (3.0 log_10_ at day 211) following an ALT flare. Given that reductions in absolute HBsAg levels were observed in some HBeAg-positive patients with high baseline HBsAg, exploration of a longer treatment duration may improve the response in HBeAg-positive patients.

Three patients treated with bepirovirsen 300 mg had a minimal response with <0.2 log_10_-transformed reduction in HBsAg. The first patient was treatment-naïve and HBeAg positive, with a high baseline HBsAg (5.04 log_10_ IU ml^−1^). Although only achieving <0.2 log_10_-transformed reduction, HBsAg levels decreased from ~110,000 IU ml^−1^ at day 1 to ~81,000 IU ml^−1^ at day 36. It is possible that the duration of exposure to bepirovirsen was not long enough to elicit a robust response. The second and third patients were treatment-naïve and on-NA treatment, respectively. Both were HBeAg negative, and both had a baseline HBsAg level similar to patients with a robust response, indicating that baseline HBsAg levels do not solely explain the lack of response. Additionally, sequencing of samples from both patients confirmed the presence of the bepirovirsen binding site, indicating that the less robust HBsAg decrease is not due to loss of the target site. Despite the minimal reduction in HBsAg, the treatment-naïve patient had a –0.81 log_10_-transformed reduction in HBV DNA at day 29, indicating that HBV in this patient was possibly susceptible to bepirovirsen; however, this patient’s HBV DNA levels were already declining from screening. The HBV RNA and HBcrAg were both at the LLOQ at baseline and throughout the study. The on-NA patient had detectable HBcrAg levels at baseline, which did not change on treatment; HBV DNA and RNA were not detectable at baseline. Study of bepirovirsen treatment of longer duration is needed to determine whether the HBsAg levels in such HBeAg-negative patients can be further reduced.

Previously, Wooddell et al. proposed that the less-efficient HBsAg reduction following treatment with a small interfering RNA in HBeAg-negative HBV-infected chimpanzees was due to loss of the target binding site in mRNA derived from integrated HBV genetic material; the bepirovirsen binding site is ~200 nucleotides upstream of the integration site identified by Wooddell et al.^15^. In this study, three on-NA patients (all HBeAg negative) experienced reductions in HBsAg ≥3.0 log_10_ IU ml^−1^. Baseline levels of HBcrAg and HBV RNA were at or near to the limit of assay sensitivity, which limits interpretation, but implies that low levels of covalently closed circular HBV DNA transcriptional activity are present and most HBsAg is likely derived from the integrated DNA. This suggests that bepirovirsen can reduce transcripts derived from integrated HBV DNA.

As expected, HBV DNA levels declined substantially in treatment-naïve patients after administration of NA treatment on day 29. These reductions were similar across the placebo, bepirovirsen 150 mg and 300 mg groups, which suggests that bepirovirsen treatment does not affect response to NAs. Furthermore, the increases in HBsAg after day 29, alongside the reductions in HBV DNA, confirm that NA treatment does not directly reduce HBsAg. However, we cannot exclude the possibility of a subtle effect early in NA treatment that may have contributed to further small reductions in HBsAg in the weeks immediately following day 29 in some patients.

Changes in ALT were assessed due to the known temporal link to HBsAg clearance, wherein ALT flares during NA therapy were associated with subsequent HBsAg decline and HBsAg loss^17^. In the current study, HBsAg reduction either preceded or occurred in parallel with the ALT increase. The temporal profile observed in this study is different from that shown in a phase 3 study of HBeAg-positive patients treated with NA in which ALT flare occurred first, with HBsAg loss occurring a median of 56 weeks later^17^. The difference may be due to the differing mechanisms of action. It was hypothesized that ALT flare following NA treatment achieved HBV control, with eventual HBsAg loss occurring via hepatocyte turnover^17^. Based on the findings of the current study, we suggest that the observed HBsAg reduction is related to bepirovirsen treatment, with the reduction in HBsAg leading to ALT increase, which is possibly due to immune clearance of infected hepatocytes. ALT increases were also observed in three on-NA patients in whom HBV DNA was suppressed already. Therefore, it is unlikely that there is a connection between HBV DNA reduction and ALT increase.

Eleven of 23 patients with CHB who received bepirovirsen experienced a maximum ALT >2× ULN during this study. By contrast, none of the 12 healthy volunteers in the phase 1 study who received a single dose of bepirovirsen 300 mg and only one of nine healthy volunteers who received bepirovirsen 300 mg for 4 weeks reported ALT increases ≥2× ULN; the patient in the bepirovirsen 300 mg arm had two ALT elevations that reached 2.6× ULN (unpublished data). Furthermore, no ALT elevations were observed in patients without a HBsAg reduction. Taken together, these results are more consistent with ALT increase as a pharmacological response to bepirovirsen targeting HBV RNA than with ALT as a toxicity from bepirovirsen exposure. However, given the small safety database, further characterization of ALT flares is warranted in future studies to further evaluate this hypothesis and rule out the possibility of drug-induced liver injury. Future studies should also consider the potential for grade 4 ALT flares in patients with cirrhosis, who were excluded in this study. A cautious approach is needed in these patients to minimize the risk from ALT elevations in patients with impaired hepatic reserve.

The observed transient appearance of anti-HBs antibody in some patients is consistent with the continuous synthesis of antibody that is sequestered into immune complexes by a large excess of circulating HBsAg. Reducing HBsAg via treatment with bepirovirsen changes this equilibrium so that free antibody appears in the circulation; the gradual increases in HBsAg observed in most patients after the end of bepirovirsen treatment may reduce circulating anti-HBs antibodies again via sequestration.

Bepirovirsen showed an acceptable safety and tolerability profile in patients with CHB. It is well established that adverse events (AEs) can lead to low clinical adherence^9^, and this is an important consideration given that the participants receiving bepirovirsen experienced more AEs of fever and injection site conditions. However, these events were mostly grade 1, and did not require dose interruptions. Given the generally mild nature of these AEs, and the overall safety profile, bepirovirsen was considered suitable for continued clinical study; however, longer studies are required to provide more insight into the tolerability of and adherence to bepirovirsen when administered for longer periods. There were no serious AEs other than the ALT increases discussed above. CRP elevations were observed in patients with CHB, which is in line with previous studies of phosphorothioate oligonucleotides^18–20^, suggesting that it may be a class effect. Complement activation is an established class effect in nonhuman primates, although this has not translated into humans^21^; the lack of complement activation related to bepirovirsen is consistent with previous clinical studies of phosphorothioate oligonucleotides. Similarly, dose-related, transient activated partial thromboplastin time prolongations following administration of bepirovirsen were observed and are a known class effect of phosphorothioate oligonucleotides^22,23^.

The limitations of this study include small patient numbers and the short duration of bepirovirsen treatment, which preclude a conclusion of whether bepirovirsen can achieve functional cure. Although the 150 mg dose did not result in a statistically significant reduction in HBsAg, it merits consideration as part of a consolidation regimen. Additionally, immunological investigations will be important to evaluate the hypothesis of immune restoration indicated by ALT flares associated with HBsAg reductions. Furthermore, the study did not provide insight into effects in the liver due to the absence of liver biopsies.

In summary, this study suggests that bepirovirsen can induce rapid and prolonged reductions in HBsAg in patients with CHB, both treatment-naïve patients and those on stable NA therapy. Combined with a favorable safety profile, these preliminary findings warrant further investigation into the dose and duration of bepirovirsen in a larger population of patients with CHB.

## Methods

### Study design

The study was approved by an independent ethics committee or institutional review board (IRB): IRB of the University of Hong Kong/Hospital Authority Hong Kong West Cluster, Queen Mary Hospital, Hong Kong; Seoul National University Hospital IRB, Republic of Korea; Kyungpook National University IRB, Republic of Korea; Seoul St. Mary’s Hospital IRB Republic of Korea; Pusan National University Hospital IRB, Republic of Korea; Korea University Ansan Hospital IRB, Republic of Korea; Inje University Busan Paik Hospital IRB Republic of Korea. The study was conducted in accordance with the Declaration of Helsinki (https://www.wma.net/policies-post/wma-declaration-of-helsinki-ethical-principles-for-medical-research-involving-human-subjects/) and International Conference on Harmonisation Good Clinical Practice (https://ichgcp.net/). An independent monitoring committee oversaw the study. All patients provided written informed consent to participate in the trial before any study-specific procedures; patients were not compensated for participation in the study, but where permitted by local regulations and ethics committees, reasonable expenses were reimbursed.

The trial was composed of a double-blinded, randomized, placebo-controlled, dose-escalation study in treatment-naïve patients (*n* = 24) and an add-on exploratory cohort of patients already receiving stable NA regimens (on-NA patients; *n* = 7) (Supplementary Fig. 1). Patients were enrolled from one center in Hong Kong and five centers in the Republic of Korea. The first patient was enrolled on 22 February 2017, the last patient was enrolled on 30 April 2019, the last patient visit was on 18 December 2019 and the study was completed on 19 December 2019. Patients were identified by the investigators or enquired about the study after reading patient information materials. The demography of the patients is broadly consistent with the HBV patient population.

The rationale for the weekly dosing frequency was supported by prior preclinical and clinical evaluations of more than 15 other 2′-MOE chimeric ASOs administered during preclinical and clinical studies^24,25^. All these 2′-MOE chimeric ASOs have very similar physico-chemical properties, because they have largely similar sugar–phosphorothioate–sugar backbones, and vary mainly in the order of the four bases^24,25^. Twice weekly dosing in weeks 1 and 2 constituted loading doses, with the aim of reaching steady-state hepatic concentrations following the week 3 dose (that is, before the last dose in the schedule) instead of 13–15 weeks without loading. The main assessments of single-agent treatment effects of bepirovirsen were performed on day 29, 7 d after the last dose of study drug (bepirovirsen or placebo). After these assessments, all patients received daily treatment with NA to end of study (week 31; day 211). Patients already on a stable NA regimen (on-NA) received treatment throughout (during all study periods).

### Objectives and endpoints

The primary objective of the study was to examine the safety and tolerability of bepirovirsen administration in treatment-naïve participants with CHB infection (primary endpoints were AEs, clinical laboratory tests, vital signs and body weight, physical examination, electrocardiogram and concomitant medication usage).

Secondary objectives and endpoints were to: examine the effects of bepirovirsen administration on plasma HBV DNA concentration (change from baseline to day 29 and week 31); examine the effects of bepirovirsen administration on serum HBsAg concentration (change from baseline to day 29 and week 31, proportion of participants with HBsAg loss at day 29 and at week 31); examine the effect of bepirovirsen administration on serum HBeAg concentration in patients who were HBeAg positive at baseline (change from baseline to day 29 and week 31, proportion of participants with HBeAg loss at day 29 and at week 31); assess plasma pharmacokinetics of bepirovirsen in patients with chronic HBV infection (to be published separately); and describe the safety and tolerability of tenofovir disoproxil fumarate (TDF) (and entecavir (ETV) if administered) therapy following conclusion of bepirovirsen administration (AEs after day 29; as the safety profile of TDF and ETV is well established we have not reported this endpoint here).

Exploratory endpoints and objectives included describing the rate of seroconversion to anti-HBs or anti-HBe antibody-positive during treatment with bepirovirsen and then during subsequent treatment with TDF, or ETV if administered (proportion of patients with antibody positivity at day 29 and at week 31).

### Key eligibility criteria

#### Inclusion criteria

Inclusion criteria were chronic HBV infection ≥6 months and serum HBsAg ≥50 IU ml^−1^; both HBeAg-positive and HBeAg-negative patients could participate. Treatment-naïve patients had a plasma HBV DNA ≥2 × 10^3^ IU ml^−1^. On-NA patients had HBV DNA adequately suppressed (plasma or serum HBV DNA below LLOQ (20 IU ml^−1^)), were taking stable TDF or ETV for ≥12 months and expected to continue taking stable TDF or ETV without change through to the end of their participation in this study.

#### Exclusion criteria

Exclusion criteria were: a history of liver cirrhosis and/or evidence of cirrhosis, liver failure, liver disease other than hepatitis B, Gilbert’s syndrome or history of laboratory results consistent with Gilbert’s syndrome, extrahepatic disorders possibly related to HBV immune complexes, excess alcohol consumption; co-infection with hepatitis C virus, hepatitis D virus or HIV; screening laboratory values of ALT and AST >5× ULN. Treatment-naïve patients were in current or prior receipt of anti-HBV NA therapy. Patients who had failed prior interferon treatment more than 6 months before screening may be evaluated for possible participation in the study.

Full eligibility criteria are listed in Supplementary Methods.

### Treatment and follow-up schedule

This study was composed of a double-blinded, randomized, placebo-controlled, dose-escalation study in treatment-naïve patients with CHB. Escalation to the next dose level required Data and Safety Monitoring Board approval. Dose escalation and study enrollment ceased in the event of death of one patient or the occurrence of the same dose-limiting toxicity in two patients within one dose cohort. Dose-limiting toxicity was defined as AEs possibly related or related to study drug administration that met the following criteria: confirmed laboratory result meeting one of the stopping criteria (Supplementary Methods); all DAIDS grade ≥3 nonlaboratory AEs with the exception of influenza-like symptoms consistent with the typical acute, transient, responses to 2′-MOE-modified chimeric phosphorothioate ASO injection; or any DAIDS grade 4 confirmed laboratory AE (other than those included in the stopping criteria; Supplementary Methods).

Although the Data and Safety Monitoring Board supported dose escalation to 450 mg, it was acknowledged that further evaluation of the 300 mg dose was warranted and the sponsor continued evaluation of the 300 mg dose in the third cohort to allow further characterization of the antiviral effect of bepirovirsen at the 300 mg dose level. One exploratory cohort was added to evaluate add-on treatment with bepirovirsen 300 mg versus placebo in on-NA patients with CHB. Approximately eight patients were planned. The study design is illustrated in Supplementary Fig. 1.

Patients were randomized (3:1 within each dose cohort) to bepirovirsen or placebo according to the randomization schedule (permuted block). The investigator (or designee) obtained the unique study treatment number via an interactive voice/internet response system. Six doses of bepirovirsen or placebo were administered via subcutaneous injection on days 1, 4, 8, 11, 15 and 22; patients were followed until day 211. On day 29, the effects of treatment were assessed. The endpoint assessment for antiviral activity was on day 29. TDF (or ETV) treatment was initiated on day 29 after HBV measurements in the treatment-naïve cohort.

All participants, study monitors, study center personnel and contract research organization personnel were blinded to treatment assignment. A global protocol amendment was made to include an on-NA cohort, in which participants with CHB already on a stable regimen of TDF or ETV were treated with 300 mg of bepirovirsen by the same dosing schedule as treatment-naïve patients; all applicable sections of the protocol (including inclusion and exclusion criteria) were updated to reflect the addition of the on-NA cohort. The amendment, implemented after study initiation and demonstration of the drug’s antiviral effect in treatment naïve patients, was introduced to determine whether antiviral effects could be observed in patients receiving stable NA therapy. The 300 mg dose of bepirovirsen was selected for this cohort based on the data from the treatment-naïve patient cohort. All other protocol amendments and changes to study conduct or planned analyses are described in Supplementary Methods. All protocol amendments were approved by the designated IRB for each study site (Methods).

### Assessments

Data were collected from 22 February 2017 to 19 December 2019. Blood samples for quantitative HBsAg, HBeAg and HBV DNA measurement were collected at screening, pre-dose on days 1, 15 and 29, and any time on days 23, 36, 57, 85, 113 and 211. Blood samples for categorical anti-HBs and anti-HBe antibodies measurement were collected at screening, pre-dose on days 1 and 29, and any time on days 57, 113 and 211.

Virology assessments included quantitative measurement of serum HBsAg (COBAS HBsAg quant II; LLOQ: 0.05 IU ml^−1^ (Roche)), serum HBV DNA (COBAS Ampliprep/COBAS Taqman HBV test v.2.0 (Roche); LLOQ: 20 IU ml^−1^), serum HBeAg (LIAISON quantitative hepatitis B antigen by electrochemiluminescence immunoassay (DiaSorin)), serum HBcrAg (Lumipulse chemiluminescent enzyme immunoassay (Fujirebio), which utilized the Lumipulse G series system; LLOQ: 3.0 log_10_ U ml^−1^), HBs antibodies (COBAS Elecsys anti-HBs II (Roche)), and HBe antibodies (COBAS Elecsys anti-HBe ECLIA (Roche)). Levels of HBV RNA were assayed by quantitative real-time polymerase chain reaction in the Applied Biosystems 7900HT Fast Real-Time PCR System with a TaqMan probe method (LLOQ: 10,862 copies ml^−1^). Population sequencing of HBV DNA or RNA was carried out on samples collected at baseline, end of bepirovirsen treatment period (day 29) and at day 113.

HBsAg and HBeAg quantitation were performed by PPD laboratories. HBV RNA quantitation was performed at DDL Diagnostic Laboratory. DNA and RNA sequencing were performed by WuXi AppTec to detect the presence of the bepirovirsen binding site sequence (GCACTTCGCTTCACCTCTGC).

### Statistical methods

The enrolled population included all patients who signed the informed consent form. The safety population included all randomized patients who received one or more doses of bepirovirsen or placebo and was used for all safety analyses. The full analysis population (representing the practically feasible intent-to-treat population) included patients from the safety population with baseline and one or more post-baseline plasma HBV DNA concentrations. The per-protocol population included patients in the full analysis set who received at least five doses of bepirovirsen or placebo during the 4-week treatment period, had plasma HBV DNA concentration measured at day 29, and had no significant protocol deviations that would be expected to affect efficacy assessments. The full analysis and per protocol populations were used for efficacy endpoints.

There is no statistical rationale for the selected sample size of eight participants per cohort. The sample size was based on prior experience to ensure adequate initial assessment of the safety and tolerability of bepirovirsen while minimizing the number of patients unnecessarily exposed to the drug. The protocol included the ability to repeat a cohort if additional data at a specified cohort was warranted. No power calculation was performed.

No formal interim analysis was planned. However, an unblinded interim analysis for treatment-naïve dose cohorts was performed after all treatment-naïve patients had completed their day 29 assessments. An unblinded interim analysis for on-NA patients was performed after all on-NA patients had completed their day 29 assessments.

SAS v.9.4 was used for data analyses in this study. Demographic and baseline characteristics, and efficacy endpoints were summarized descriptively. Safety analyses were conducted on the safety population and summarized by treatment group. There were no formal hypotheses; efficacy analyses for comparison between bepirovirsen and the pooled placebo group were performed in an exploratory manner. Changes from baseline to day 29 or week 31 for plasma HBV DNA, serum HBsAg and serum HBeAg concentrations were logarithmic transformed with base 10. The LOCF method was used to impute missing values. Comparison between bepirovirsen and a pooled placebo group was performed separately for each dose level using an analysis of covariance model with baseline as a covariate and treatment group as a factor. The proportion of participants with reduction in plasma HBV DNA, serum HBsAg and serum HBeAg concentrations of at least 0.5, 1.0, 1.5 and 2.0 log_10_ at day 29 and week 31 were conducted; comparison between bepirovirsen and placebo was performed using Fisher’s exact test. All statistical tests were conducted using two-sided tests with 5% type I error rates unless otherwise stated. Summaries of HBV RNA and HBcrAg were conducted post hoc.

### Reporting Summary

Further information on research design is available in the Nature Research Reporting Summary linked to this article.

## Online content

Any methods, additional references, Nature Research reporting summaries, source data, extended data, supplementary information, acknowledgements, peer review information; details of author contributions and competing interests; and statements of data and code availability are available at 10.1038/s41591-021-01513-4.

## Supplementary information

Supplementary Information Supplementary Methods, Results, Figs. 1–10 and Table 1.

Reporting Summary

## Data Availability

Within 6 months of this publication, anonymized individual participant data, the annotated case report form, protocol, reporting and analysis plan, dataset specifications, raw dataset, analysis-ready dataset and clinical study report will be available for research proposals approved by an independent review committee. Proposals should be submitted to www.clinicalstudydatarequest.com. A data access agreement will be required.
