# Supplementary material for: Safety, tolerability and antiviral activity of the antisense oligonucleotide bepirovirsen in patients with chronic hepatitis B: a phase 2 randomized controlled trial
Source: Nat Med. 2021 Oct 12;27(10):1725–34. doi: 10.1038/s41591-021-01513-4 (PMC8516644; doi:10.1038/s41591-021-01513-4)
Supplement: Supplementary file 1 — Supplementary Methods, Results, Figs. 1–10 and Table 1. [file 41591_2021_1513_MOESM1_ESM.pdf]

nature portfolio
https://doi.org/10.1038/s41591-021-01513-4

Supplementary information

Safety, tolerability and antiviral activity of the antisense oligonucleotide bepirovirsen in patients with chronic hepatitis B: a phase 2 randomized controlled trial

In the format provided by the authors and unedited

SUPPLEMENTARY MATERIAL

Supplementary methods

Full eligibility criteria

Inclusion criteria

1. Have given written informed consent (signed and dated) and any authorizations required by local law and is able to comply with all study requirements
2. Age 18–70 years
3. Chronic HBV infection ≥6 months (e.g., positive for serum hepatitis B surface antigen [HBsAg] ≥6 months)
4. Treatment-naïve: Plasma HBV DNA $\geq 2\times10^3$ IU/mL; patients already receiving stable nucleos(t)ide analog (NA) regimens (on-NA); HBV DNA adequately suppressed (e.g., plasma or serum HBV DNA below the lower limit of quantitation)
5. Serum HBsAg >50 IU/mL
6. Satisfy the following:
    a. Females: Non-pregnant and non-lactating; surgically sterile (e.g., tubal occlusion, hysterectomy, bilateral salpingectomy, bilateral oophorectomy), post-menopausal (defined as 12 months of spontaneous amenorrhea in females >55 years of age or, in females ≤55 years, 12 months of spontaneous amenorrhea without an alternative medical cause and FSH levels in the postmenopausal range for the laboratory involved) or, if engaged in sexual relations of child-bearing potential, patient is using an acceptable contraceptive method from the time of signing the informed consent form until at least 13 weeks after the last dose of study drug

    b. Males: Surgically sterile or if engaged in sexual relations with a female of childbearing potential, patient is utilizing an acceptable contraceptive method during treatment with study drug and for at least 13 weeks after the last dose of study drug

7. On-NA only: Currently taking and have been taking tenofovir or entecavir without changes in drug, dose level, and/or frequency of administration for ≥12 months and expect to continue taking without change through to the end of their participation in this study (approximately 8 months)

Exclusion criteria

1. Treatment-naïve only: Current or prior receipt of anti-HBV NA therapy. Patients who have failed prior interferon treatment, greater than 6 months prior to screening, may be evaluated for possible participation in the study.
2. History of liver cirrhosis and/or evidence of cirrhosis as determined by any 1 of the following:
    a. Liver biopsy (i.e., Metavir Score F4) within 2 years of screening, or
    b. FibroScan (echosens, Paris, France) > 12 KPa, within 12 months of screening, or
    c. AST-to-Platelet Index (APRI) > 2 and FibroSure (LabCorp, Burlington, USA) result > 0.7 within 12 months of screening

    For patients without a test for cirrhosis in the above timeframes, FibroScan (echosens, Paris, France), or APRI and FibroSure (LabCorp, Burlington, NC, USA), may be performed during the screening period to rule out cirrhosis
3. History of liver failure as evidenced by ascites, hepatic encephalopathy, and/or gastric or esophageal varices
4. History of liver disease other than hepatitis B
5. Co-infection with HCV, HIV, or hepatitis D virus (HDV)

6. Body mass index >35 kg/m^2
7. History or suspected presence of vasculitis
8. Received solid organ or bone marrow transplant
9. Currently taking, or took within 3 months of screening, any immunosuppressing drugs (e.g., prednisone). If the patient received a short course (≤1 week), the situation may be discussed with the medical monitor, or designee
10. Diagnosed hepatocellular carcinoma or suspected hepatocellular carcinoma as evidenced by screening alpha-fetoprotein ≥200 ng/mL. If the screening alpha-fetoprotein is ≥50 ng/mL and <200 ng/mL, the absence of liver mass must be documented by imaging within 6 months before randomization
11. Screening laboratory results as follows, or any other clinically significant abnormalities in screening laboratory values that would render a patient unsuitable for inclusion
    a. ALT or AST >5 x ULN
    b. Total bilirubin >1.1 x ULN
    c. Serum albumin <3.5 g/dL
    d. International normalized ratio >1.2
    e. Platelet count <140 k/mm^3
    f. Hemoglobin <12.0 g/dL for males and <11.0 g/dL for females
    g. White blood cell count <3.0 k/mm^3
    h. Serum creatinine >1.1 x ULN
    i. Urine protein/creatinine ratio ≥0.2 mg/mg. In the event of a ratio above this threshold, eligibility may be confirmed by a quantitative total urine protein measurement of <150 mg/24 hour

j. Positive test (including trace) for blood on urinalysis. In the event of a positive test, eligibility may be confirmed with urine microscopy showing <5 red blood cells per high power field

12. History of Gilbert's Syndrome or history of laboratory results consistent with Gilbert's Syndrome
13. Clinically significant abnormalities aside from chronic HBV infection in medical history (e.g., previous acute coronary syndrome within 6 months of screening, major surgery within 3 months of screening, uncontrolled diabetes) or physical examination
14. History of bleeding diathesis or coagulopathy
15. History of extrahepatic disorders possibly related to HBV immune complexes (e.g., glomerulonephritis, polyarteritis nodosa)
16. Active infection other than HBV requiring systemic antiviral or antimicrobial therapy that will not be completed prior to Study Day 1
17. Malignancy within 5 years, except for basal or squamous cell carcinoma of the skin or carcinoma in situ of the cervix that has been successfully treated
18. Treatment with another investigational drug, biological agent or device within 4 weeks of screening or 5 half-lives of study drug, whichever is longer
19. Treatment with any oligonucleotide (not developed by ISIS Pharmaceuticals, Inc.; including siRNA) at any time
20. Prior treatment with an oligonucleotide (developed by ISIS Pharmaceuticals, Inc.) within 9 months of screening
21. History of excess alcohol consumption within 6 months of screening as defined as weekly intake of >14 drinks per week (>2 drinks per day) for males or >7 drinks per week (>1 drink in a day) for females (one drink is equivalent to 12 g of alcohol, e.g., 12 ounces [360 mL] of beer, 5 ounces [150 mL] of wine or 1.5 ounces [45 mL] of 80 proof distilled spirits)

22. History of drug abuse or dependence, or recreational use of drugs: within 3 months of screening for soft drugs (such as marijuana) and within 1 year of screening for hard drugs (such as cocaine, phencyclidine)
23. Unwillingness to comply with study procedures, including follow-up, as specified by this protocol, or unwillingness to cooperate fully with the investigator
24. Have any other conditions (medical, social or other), which in the opinion of the investigator would make the patient unsuitable for inclusion, or could interfere with the patient participating in or completing the study
25. Patients with hereditary problems of galactose intolerance, Lapp lactose deficiency, or glucose-galactose malabsorption, unless the patient will be treated with the lactose-free formulation of entecavir during the study

Stopping criteria

Stopping rules for liver chemistry elevations

Baseline ALT ≤2xULN:

- If confirmed ALT or AST ≥8xULN, permanently discontinue study drug
- If confirmed ALT or AST ≥3 to <8 x ULN, permanently discontinue study drug if any of the following apply:
    - Appearance or worsening of symptoms felt by the investigator to be potentially related to worsening of hepatic inflammation such as fatigue, nausea, vomiting, right upper quadrant pain or tenderness, fever, rash, and/or concomitant eosinophilia (>ULN)
    - Total bilirubin >1.5xULN and direct bilirubin >35%

Baseline ALT >2xULN:

- If confirmed ALT or AST ≥4x baseline, or confirmed ALT or AST ≥20xULN, permanently discontinue study drug
- If confirmed ALT or AST ≥2 to <4x baseline, permanently discontinue study drug if any of the following apply:
    - Appearance or worsening of symptoms felt by the investigator to be potentially related to worsening of hepatic inflammation such as fatigue, nausea, vomiting, right upper quadrant pain or tenderness, fever, rash, and/or concomitant eosinophilia (>ULN)
    - Total bilirubin >1.5xULN and direct bilirubin >35%

Note: Baseline is latest laboratory result prior to first dose of study drug

Stopping rules for hematologic test results

- Hemoglobin <9.0 g/dL (i.e., Division of Acquired Immune Deficiency Syndrome [DAIDS] Grade ≥3)
- White blood cell count <1.5 k/mm^3 (i.e., DAIDS Grade ≥3)
- Absolute neutrophil count <0.75 k/mm^3 (i.e., DAIDS Grade ≥3)
- Platelet count <75 k/mm^3

Stopping rules for renal function test results

- Confirmed serum creatinine increase that is both ≥0.3 mg/dL and ≥40% above baseline creatinine values (defined as the average of the screening and study Day 1 results) and above upper limit of the reference range (i.e., >ULN)
- Confirmed urine protein/creatinine ratio ≥0.90
- Evidence of glomerular injury on urine microscopic exam

Stopping rules for significant inflammatory reaction

An event (without a probable cause other than study drug) of clinical symptoms (e.g., constellation of symptoms such as severe fever, chills, and myalgia) or confirmed laboratory findings (e.g., substantial decrease in serum complement protein levels) consistent with a significant inflammatory reaction.

Protocol amendments

The original protocol (dated September 2, 2015) was amended three times as follows:

Global Protocol Amendment 1 (dated February 19, 2016): this amendment was implemented before study initiation and included the following changes:

- Clarified inclusion criterion #3 "chronic HBV infection ≥6 months" by adding an example of "positive for serum HBsAg ≥6 months"
- Provided further explanation about the flu-like symptom exception in the dose-limiting toxicity (DLT) criteria
- Added clarity to the role of bepirovirsen in defining a DLT. Provided further clarification regarding the potential actions of the Data and Safety Monitoring Board
- Added "evidence of glomerular injury on urine microscopic examination" criteria in the stopping rules for renal function test results
- Added stopping rules for significant inflammatory reaction
- Added albumin/creatinine ratio as part of the urinalysis

The Republic of Korea-specific Protocol Amendment 2 (dated November 22, 2016): this amendment was implemented before study initiation and included the following changes:

- Added the following exclusion criterion #25: Participants with hereditary problems of galactose intolerance, Lapp lactose deficiency, or glucose-galactose malabsorption, unless the participant will be treated with the lactose-free formulation of entecavir during the study

Changes in study conduct or planned analyses

After database lock and unblinding of the treatment assignments, the following changes occurred to the planned analyses based on the sponsor's request:

- The by-participant listings of safety data were generated for the safety population instead of the all enrolled population
- The summary table for tenofovir/entecavir exposure was revised from "Extent of Exposure" to "Extent of Expected Exposure" as there was no diary card or other clinical page to collect the daily tenofovir/entecavir administration information. The information of tenofovir and entecavir dosing was not captured as concomitant medications

Supplementary results

Presence of the binding site for bepirovirsen

Presence of the bepirovirsen binding site (**Figure S8**) in the viral genome was assessed by DNA sequencing of samples that contained ≥2,000 IU/mL of HBV DNA on Day 1, Day 29 and Day 113 (note for treatment naïve patients, NA was initiated on Day 29). For treatment-naïve patients in the bepirovirsen 150 mg arm, sequencing of bepirovirsen binding region on Day 1 and Day 29 samples was accomplished for all six patients and three patients at Day 113. For treatment-naïve patients in the bepirovirsen 300 mg arm, sequencing of Day 1 samples was achieved for 11/12 patients on Day 1; 7/12 on Day 29 and 3/12 on Day 113. For treatment-naïve patients in the placebo arm, sequencing was accomplished in all six patients on Day 1, 4/6 on Day 29 and 1/6 Day 113. No mutations in the binding site were detected.

As on-NA patients did not have sufficient HBV DNA levels for DNA sequencing, sequencing of virus pre-genomic RNA was attempted. Sequencing was accomplished for Day 1 samples from two patients, both of which showed no change in the RNA sequence where bepirovirsen was designed to bind.

Figure S1: (a) Study design. (b) Patient flow through study. [a]Although the DSMB supported dose escalation to 450 mg, further evaluation of the 300 mg dose was warranted and the sponsor continued evaluation of the 300 mg dose in the third cohort (instead of escalating to 450 mg) to allow further characterization of the antiviral effect of bepirovirsen at the 300 mg dose level. [b]Planned sample size; n=7 patients were randomized into cohort 4. [c]On-NA patients received NA treatment throughout the study. Treatment-naïve patients receiving 300 mg of bepirovirsen from cohorts 2 and 3 have been combined for presentation purposes. Placebo-treated patients from cohorts 1,2 and 3 have also been combined. DSMB, Data and Safety Monitoring Board.

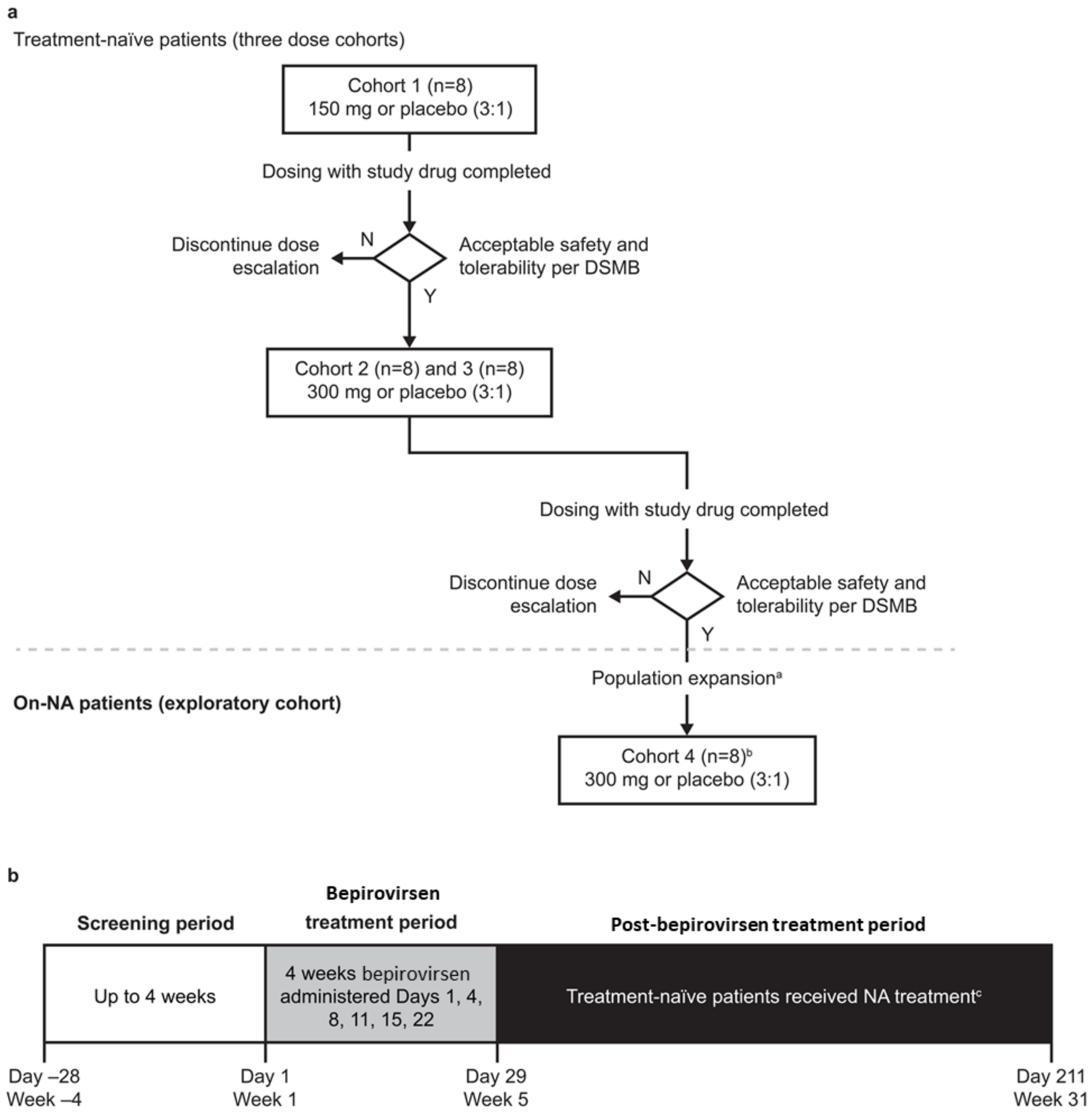
a
Treatment-naïve patients (three dose cohorts)
Cohort 1 (n=8)
150 mg or placebo (3:1)
Dosing with study drug completed
Discontinue dose escalation
N
Acceptable safety and tolerability per DSMB
Y
Cohort 2 (n=8) and 3 (n=8)
300 mg or placebo (3:1)
Dosing with study drug completed
Discontinue dose escalation
N
Acceptable safety and tolerability per DSMB
Y
Population expansion[a]
On-NA patients (exploratory cohort)
Cohort 4 (n=8)[b]
300 mg or placebo (3:1)
b
Screening period
Bepirovirsen treatment period
Post-bepirovirsen treatment period
Up to 4 weeks
4 weeks bepirovirsen administered Days 1, 4, 8, 11, 15, 22
Treatment-naïve patients received NA treatment[c]
Day –28
Week –4
Day 1
Week 1
Day 29
Week 5
Day 211
Week 31

Figure S2: Patient disposition (all enrolled population). Bepirovirsen or placebo administered by subcutaneous injection on Days 1, 4, 8, 11, 15, and 22. End of follow-up was Day 211.

ETV, entecavir; HBV, hepatitis B virus; on-NA, patients on stable nucleos(t)ide analog regimens; TAF, tenofovir alafenamide; TDF, tenofovir disoproxil fumarate. [a]The most frequent reason for screen failure in the treatment-naïve group was failure to meet inclusion/exclusion criteria (21 of 24 [87.5%] patients), and in the on-NA group was withdrawal of consent (6 of 10 [60.0%] of screen fail patients). [b]Patients receiving placebo from cohorts 1, 2 and 3 combined. [c]Patients receiving bepirovirsen 300 mg in cohort 2 and 3 combined. [d]One patient in the on-NA group (receiving TDF) withdrew during Week 1 after two doses of bepirovirsen 300 mg due to transient mild fevers after each dose (considered related to study drug). [e]All but 4 treatment-naïve patients were treated with TDF. One patient received ETV, 2 received TAF instead of TDF, and 1 received TDF for 1 week then switched to ETV due to whole-body rash (rash subsided following treatment switch).

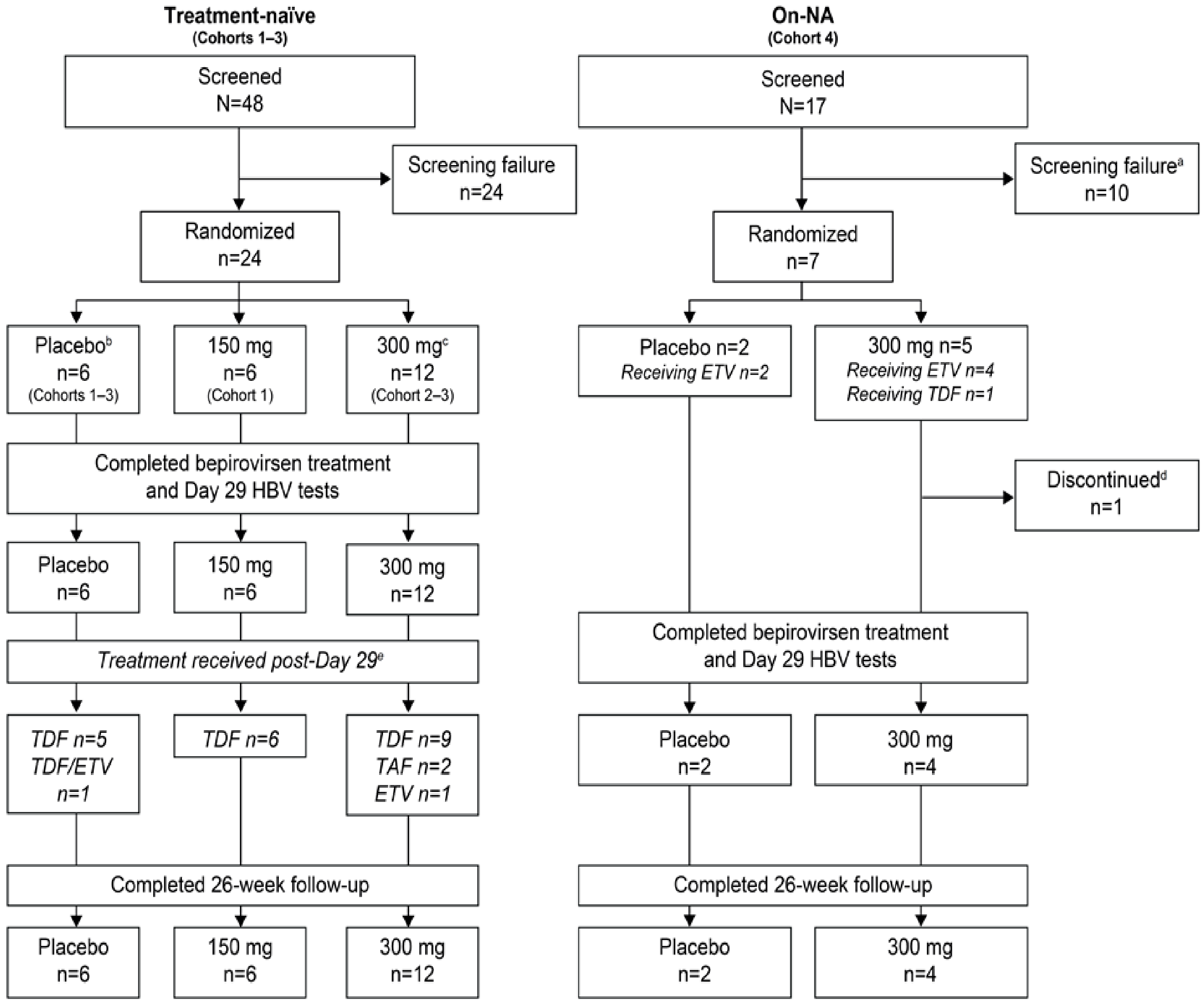
Treatment-naïve
(Cohorts 1–3)
Screened
N=48
Screening failure
n=24
Randomized
n=24
Placebo[b]
n=6
(Cohorts 1–3)
150 mg
n=6
(Cohort 1)
300 mg[c]
n=12
(Cohort 2–3)
Completed bepirovirsen treatment and Day 29 HBV tests
Placebo
n=6
150 mg
n=6
300 mg
n=12
Treatment received post-Day 29[e]
TDF n=5
TDF/ETV n=1
TDF n=6
TDF n=9
TAF n=2
ETV n=1
Completed 26-week follow-up
Placebo
n=6
150 mg
n=6
300 mg
n=12
On-NA
(Cohort 4)
Screened
N=17
Screening failure[a]
n=10
Randomized
n=7
Placebo n=2
Receiving ETV n=2
300 mg n=5
Receiving ETV n=4
Receiving TDF n=1
Discontinued[d]
n=1
Completed bepirovirsen treatment and Day 29 HBV tests
Placebo
n=2
300 mg
n=4
Completed 26-week follow-up
Placebo
n=2
300 mg
n=4

Figure S3: (a) CRP over time from an hs-CRP assay in a treatment-naïve patient treated with bepirovirsen 300 mg. Vertical lines indicate dose administration; gray shading indicates the tenofovir dosing period; horizontal dashed line indicates ULN for hs-CRP. Insert provides expanded view of Days –9 to 40. (b) hs-CRP on Day 4 for patients with CHB and Day 4 or 5 for healthy volunteers (study CS1; unpublished data), categorized by dose. Blue circles represent treatment-naïve patients; blue triangles represent on-NA patients; green circles represent Day 4 results from healthy volunteers who received single-dose bepirovirsen; green squares are Day 5 results from healthy volunteers who received multiple-dose bepirovirsen. CHB, chronic hepatitis B; CRP, C-reactive protein; HBV, hepatitis B virus; hs-CRP, high-sensitivity CRP; on-NA, patients on stable nucleos(t)ide analog regimens; ULN, upper limit of normal.

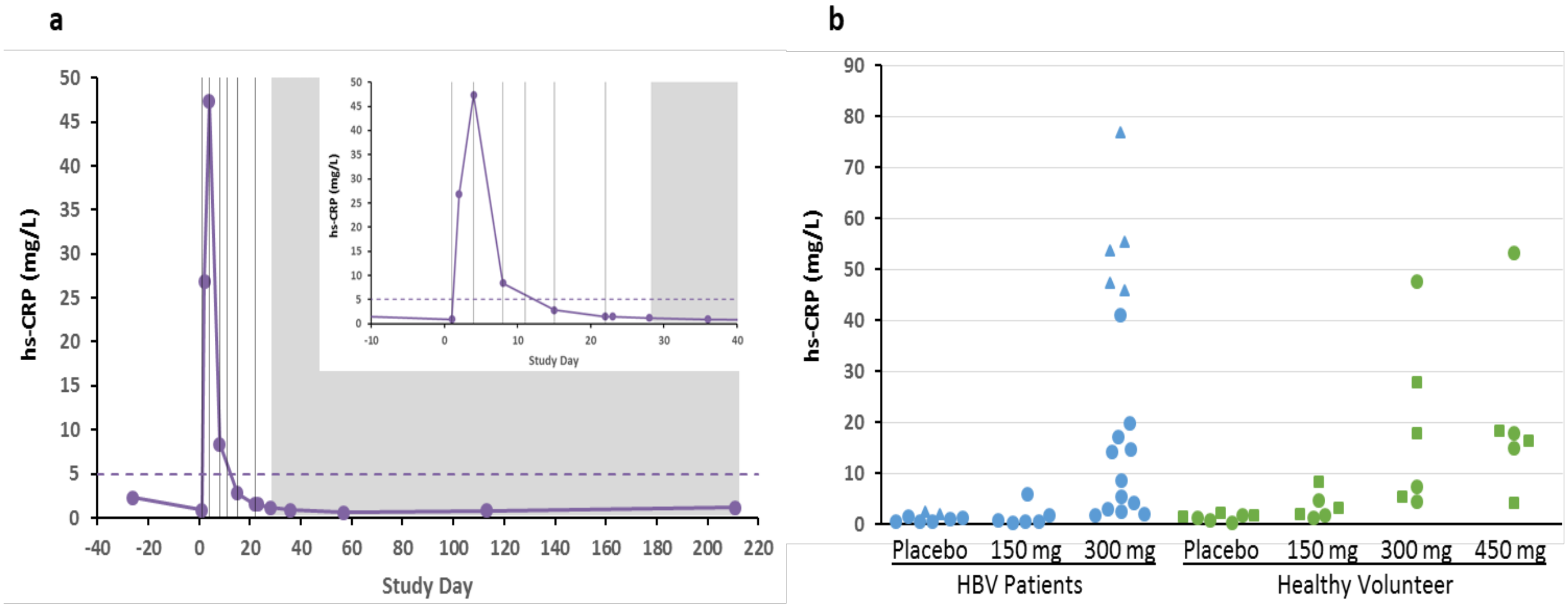

Figure S4: HBsAg and ALT over time in one HBeAg negative on-NA patient (a), one HBeAg-positive treatment naïve patient (b) and two HBeAg-negative treatment-naïve patients (c-d) with CHB treated with bepirovirsen 300 mg. Vertical lines indicate dose administration days; dashed vertical line indicates start of NA dosing period; horizontal dashed line indicates LLOQ. +, positive for anti-HBsAb; –, negative for anti-HBsAb; ±, indeterminate HBsAb status; ALT, alanine aminotransferase; CHB, chronic hepatitis B; HBeAg, hepatitis B virus e-antigen; HBsAg, hepatitis B virus surface antigen; HBV, hepatitis B virus; LLOQ, lower limit of quantitation; NA, nucleos(t)ide analog.

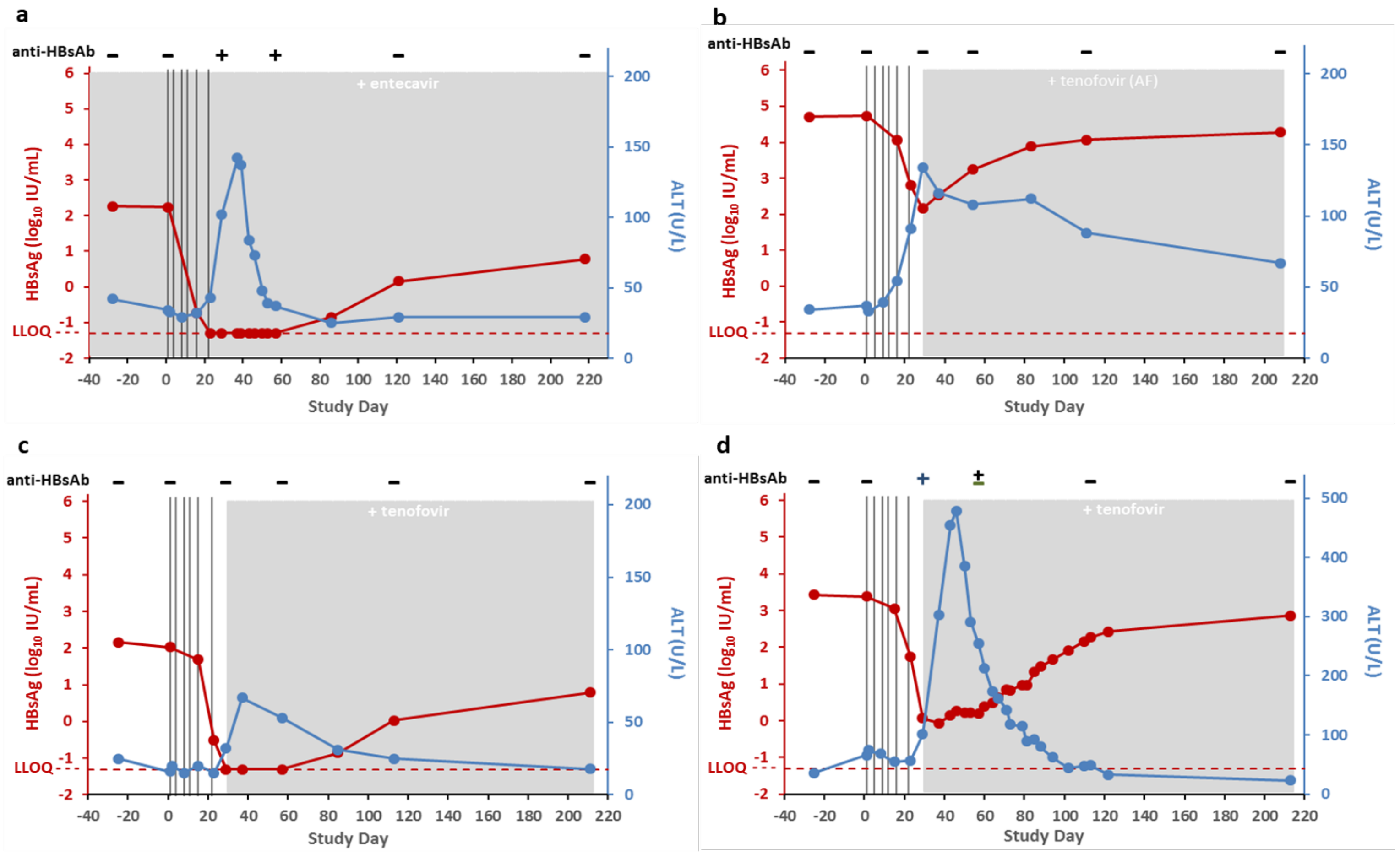

a
anti-HBsAb – – + + – –
+ entecavir
HBsAg (log10 IU/mL)
ALT (U/L)
LLOQ
Study Day
b
anti-HBsAb – – – – – –
+ tenofovir (AF)
HBsAg (log10 IU/mL)
ALT (U/L)
LLOQ
Study Day
c
anti-HBsAb – – – – – –
+ tenofovir
HBsAg (log10 IU/mL)
ALT (U/L)
LLOQ
Study Day
d
anti-HBsAb – – + ± – –
+ tenofovir
HBsAg (log10 IU/mL)
ALT (U/L)
LLOQ
Study Day

Figure S5: Individual HBsAg levels at baseline, Day 29 and Day 211 by baseline HBsAg concentration: a) 50–200 IU/mL; b) 200–1000 IU/mL; c) 1000–10,000 IU/mL; d) 10,000–40,000 IU/mL; e) 40,000 IU/mL and above. HBsAg, hepatitis B virus surface antigen; LLOQ, lower limit of quantitation.

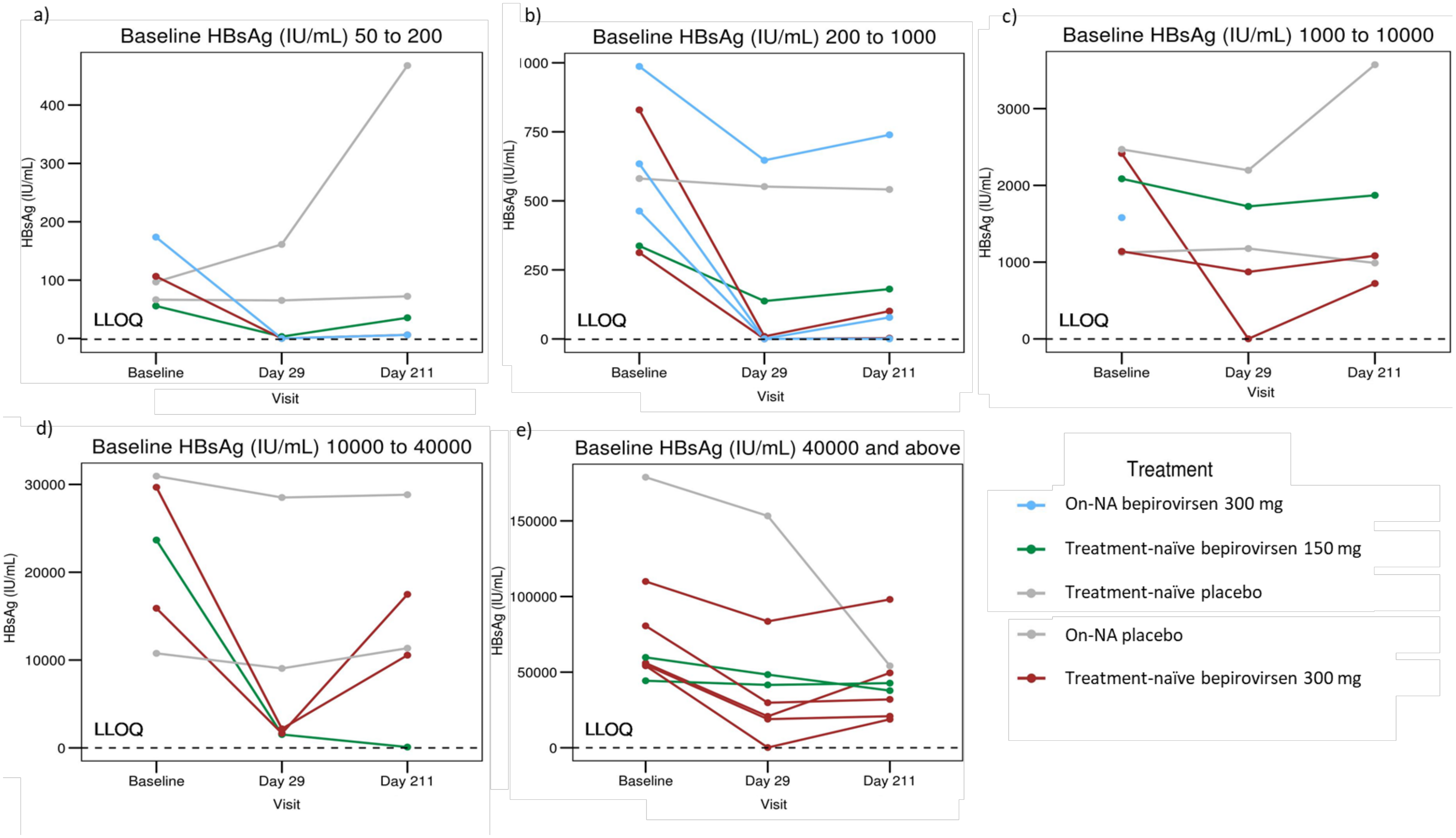

Figure S6: Reduction of a) HBcrAg, b) HBV RNA, c) HBV DNA; and d) post-baseline maximum ALT by HBsAg reduction from baseline on Day 29.

ALT, alanine aminotransferase; HBcrAg, hepatitis B core related antigen; HBsAg, hepatitis B virus surface antigen; HBV, hepatitis B virus; NA, nucleos(t)ide analog; ULN, upper limit of normal

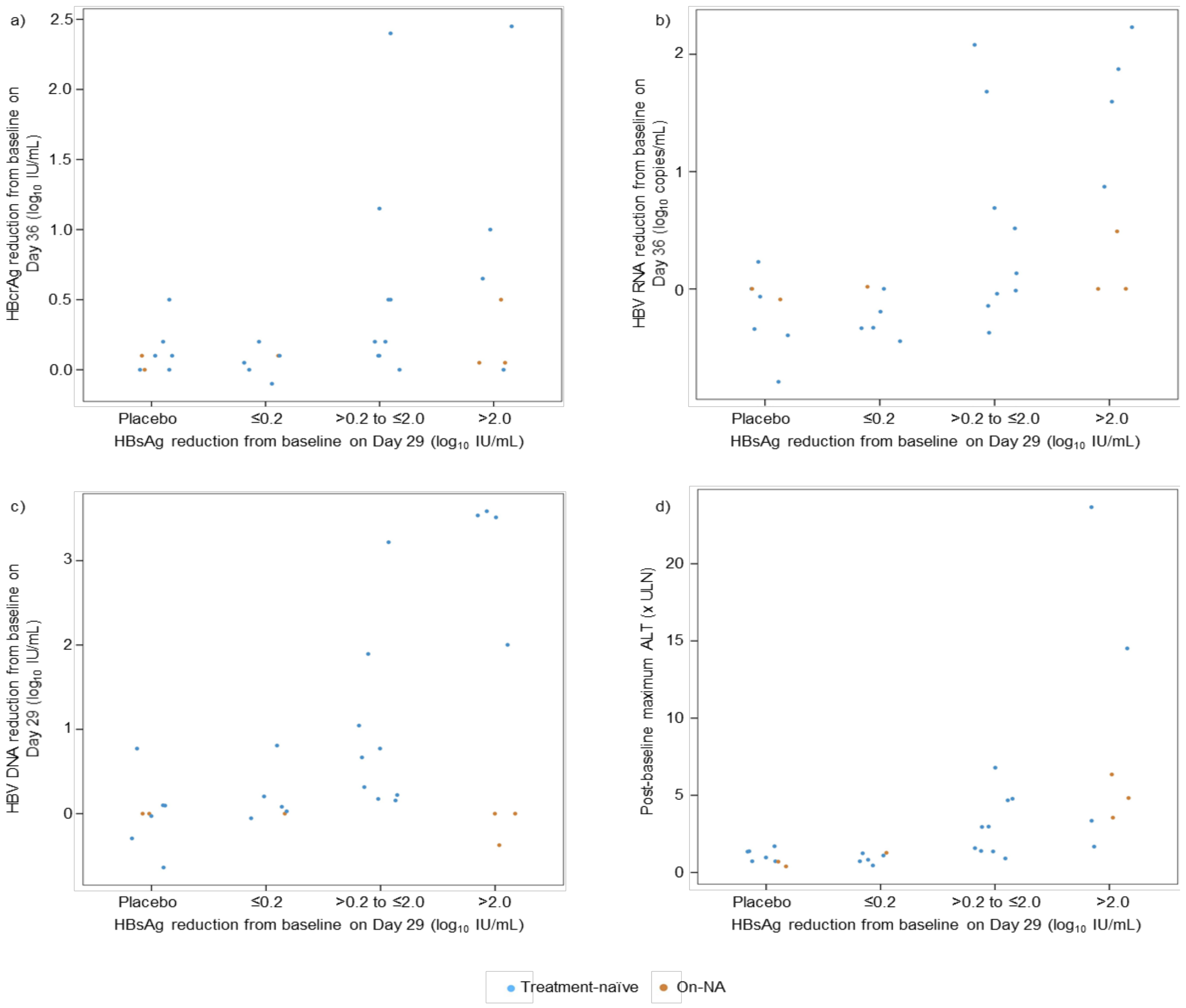

a)
HBcrAg reduction from baseline on Day 36 (log10 IU/mL)
2.5
2.0
1.5
1.0
0.5
0.0
b)
HBV RNA reduction from baseline on Day 36 (log10 copies/mL)
2
1
0
c)
HBV DNA reduction from baseline on Day 29 (log10 IU/mL)
3
2
1
0
d)
Post-baseline maximum ALT (x ULN)
20
15
10
5
0
Placebo
≤0.2
>0.2 to ≤2.0
>2.0
HBsAg reduction from baseline on Day 29 (log10 IU/mL)
Treatment-naïve
On-NA

Figure S7: Maximum ALT categorized by HBV DNA reduction from baseline; blue dots represent treatment-naïve patients with CHB (those with adjacent numbers indicate ALT flares ≥10×ULN), light brown dots represent on-NA patients with CHB. ALT, alanine aminotransferase; AUC, area under the concentration-time curve; CHB, chronic hepatitis B; HBsAg, hepatitis B virus surface antigen; HBV, hepatitis B virus; NA, nucleos(t)ide analog; ULN, upper limit of normal.

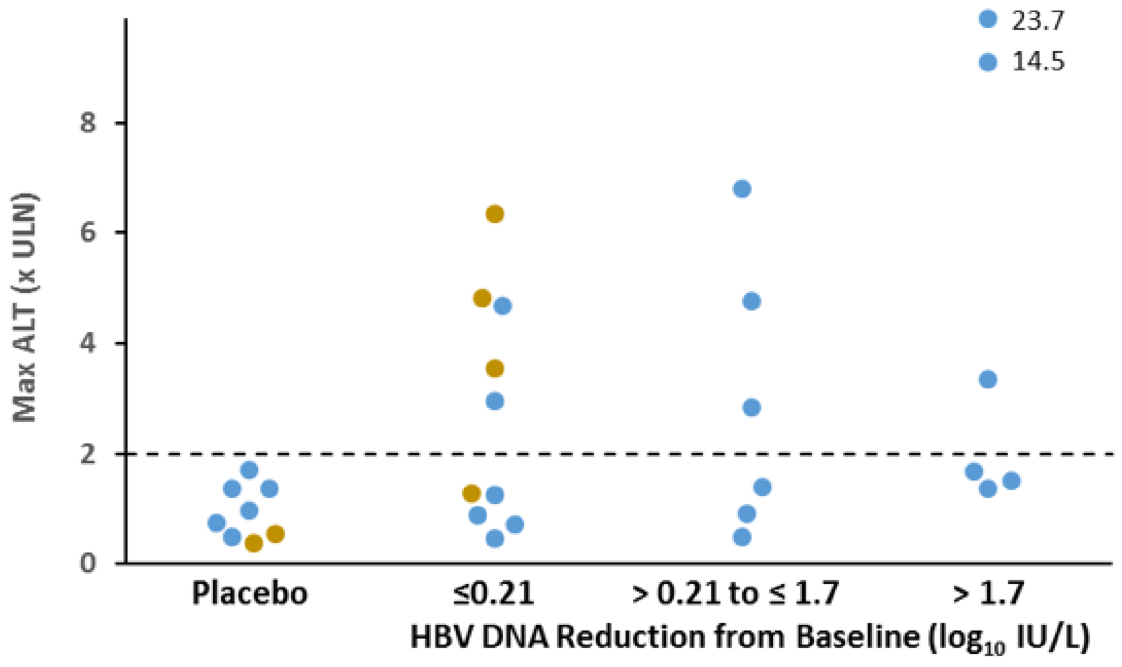

Figure S8: ALT, AST, AP, and total bilirubin over time in (a) a treatment-naïve patient in the bepirovirsen 300 mg arm with ALT flare up to 23.7 x ULN at Week 6 (recorded as an SAE), and (b) another treatment-naïve patient in the bepirovirsen 300 mg arm with ALT up to 14.5 x ULN (second highest ALT flare) at Week 6. Note: ALT flare in these patients also shown in Figure 3a and 4d, respectively. ALT, alanine aminotransferase; AP, alkaline phosphatase; AST, aspartate aminotransferase; ULN, upper limit of normal; SAE, serious adverse event; TB, total bilirubin.

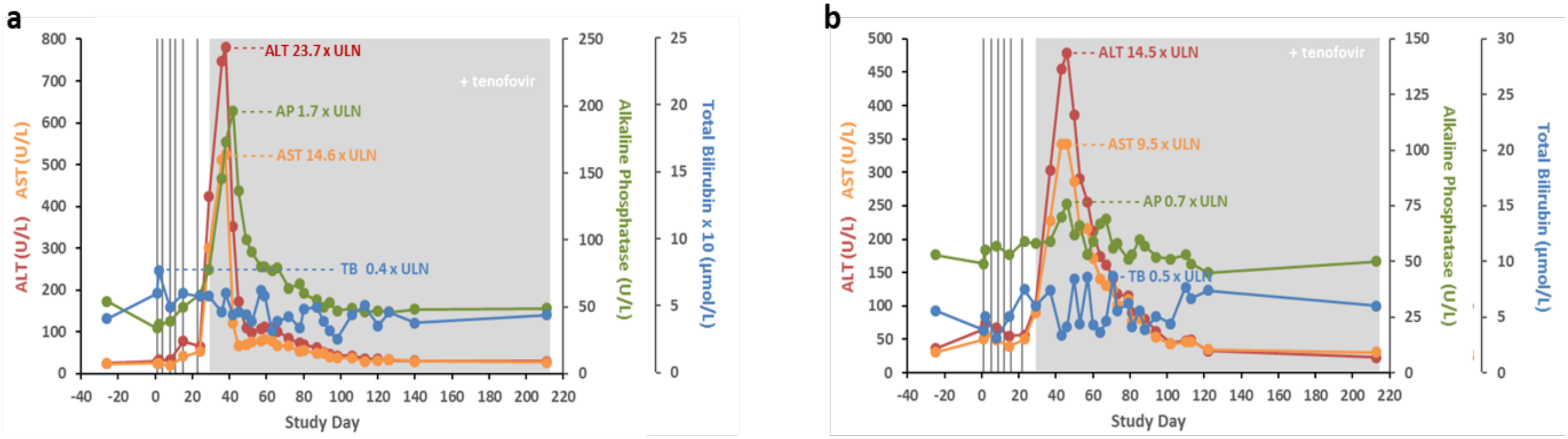

Figure S9: **(a) HBeAg and (b) HBV DNA versus ALT over time in an HBeAg-positive treatment-naïve patient in the bepirovirsen 150 mg arm.**

Note: HBsAg over time for this patient is illustrated in the main manuscript (Figure 3d). ALT, alanine aminotransferase; HBeAg, hepatitis B virus e-antigen; HBsAg, hepatitis B virus surface antigen; HBV, hepatitis B virus; LLOQ, lower limit of quantitation.

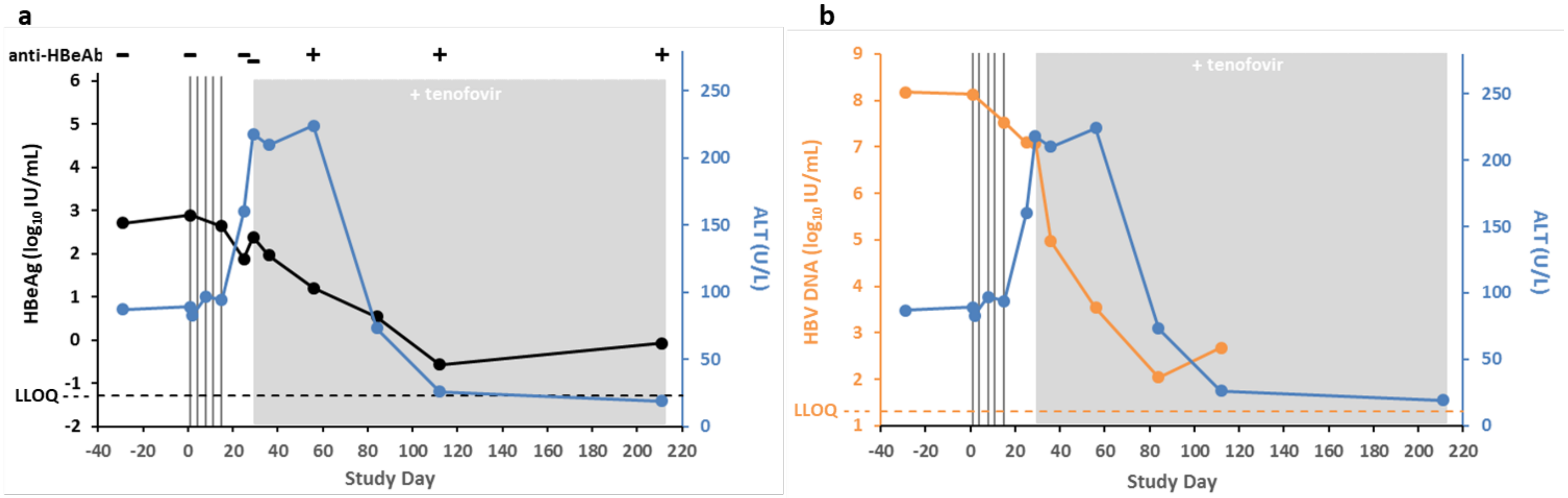

Figure S10: Bepirovirsen target site in HBV. Reprinted from Gastroenterology. 2007; 132(4). Ghany M and Liang TJ. Drug targets and molecular mechanisms of drug resistance in chronic hepatitis B. pp.1574–85, with permission from Elsevier. Bepirovirsen binding site (GCACTTCGCTTCACCTCTGC) is present in all HBV mRNA and pgRNA; HBV 11-base DR sequence is nucleotides 1–11 of bepirovirsen. DR, direct repeat; HBV, hepatitis B virus.

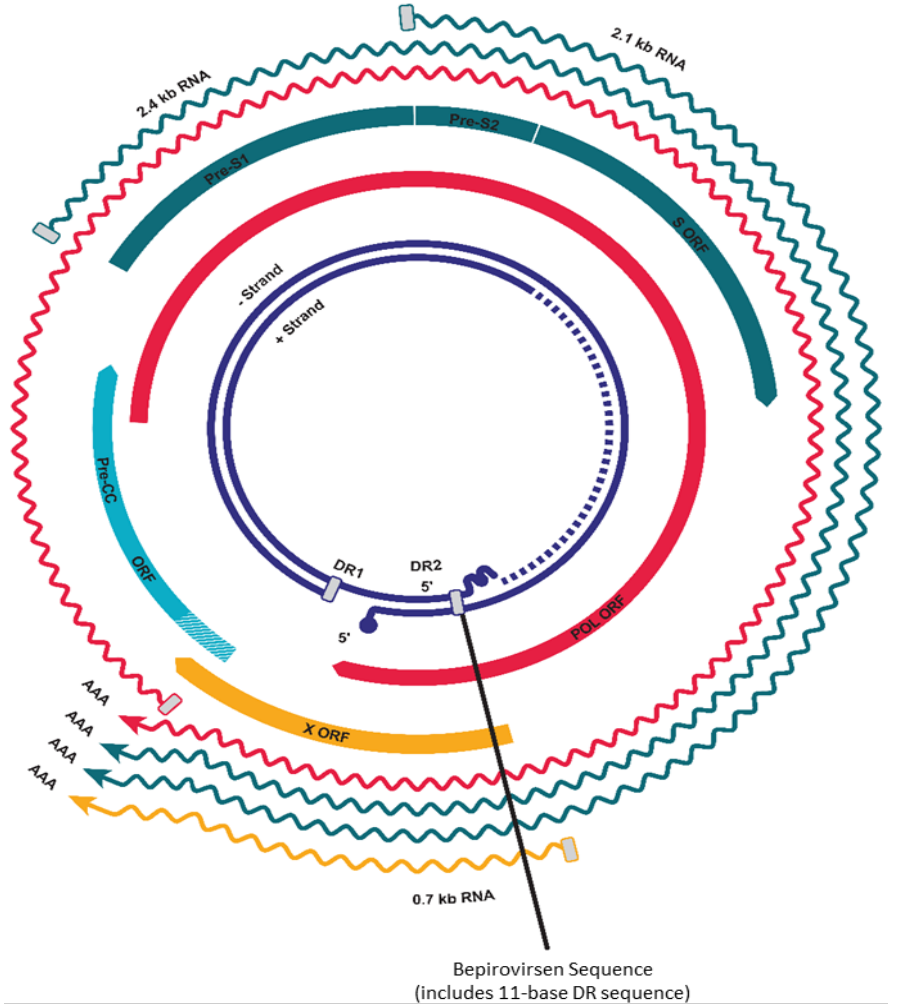

Table S1: HBeAg at baseline, Day 29 and Week 31 in treatment-naïve patients with CHB (study CS3; full analysis population).

	**Bepirovirsen 150 mg (n=6)**	**Bepirovirsen 300 mg (n=12)**	**Placebo (n=6)**
HBeAg-positive at baseline, n	5	6	2
Baseline HBeAg ($\log_{10}$ IU/mL)			
n	5	6	2
Mean (SD)	1.965 (1.3589)	2.963 (0.2818)	2.132 (0.6642)
Day 29 HBeAg ($\log_{10}$ IU/mL)			
n	5	6	2
Mean (SD)	1.961 (1.3492)	2.911 (0.7251)	2.266 (0.3758)
Change from Baseline to Day 29 in HBeAg ($\log_{10}$ IU/mL)			
n	5	6	2
Mean (SD)	–0.004 (0.3967)	–0.052 (0.6741)	0.133 (0.2884)
p-value (vs placebo)	0.763	0.804	-
Week 31 HBeAg ($\log_{10}$ IU/mL)			
n	5	6	2
Mean (SD)	1.388 (1.6078)	2.768 (0.5943)	2.263 (0.0835)
Change from baseline to Week 31 in HBeAg ($\log_{10}$ IU/mL)			
n	5	6	2
Mean (SD)	–0.577 (1.3711)	–0.195 (0.5047)	0.131 (0.7477)
p-value (vs placebo)	0.372	0.954	-

Baseline was the last non-missing measurement prior to the first dose of study drug. A patient was HBeAg positive at baseline if the baseline value was >0.09 U/mL, otherwise it was negative. Comparison between bepirovirsen and pooled placebo was performed for each dose level, separately, using an analysis of covariance (ANCOVA) model with baseline as a covariate, and treatment group as a factor. All comparisons were pre-specified with no adjustment for multiple comparisons. Two-sided p-values were presented. CHB, chronic hepatitis B; HBeAg, hepatitis B virus e antigen; HBV, hepatitis B virus; SD, standard deviation.
