# Supplementary material for: Safety, tolerability and antiviral activity of the antisense oligonucleotide bepirovirsen in patients with chronic hepatitis B: a phase 2 randomized controlled trial
Source: Nat Med. 2021 Oct 12;27(10):1725–34. doi: 10.1038/s41591-021-01513-4 (PMC8516644; doi:10.1038/s41591-021-01513-4)
Supplement: Supplementary file 2 — Reporting Summary [file 41591_2021_1513_MOESM2_ESM.pdf]

nature research

Corresponding author(s): Man-Fung Yuen

Last updated by author(s): Jul 13, 2021

Reporting Summary

Nature Research wishes to improve the reproducibility of the work that we publish. This form provides structure for consistency and transparency in reporting. For further information on Nature Research policies, see our Editorial Policies and the Editorial Policy Checklist.

Please do not complete any field with "not applicable" or n/a. Refer to the help text for what text to use if an item is not relevant to your study.
For final submission: please carefully check your responses for accuracy; you will not be able to make changes later.

Statistics

For all statistical analyses, confirm that the following items are present in the figure legend, table legend, main text, or Methods section.

n/a	Confirmed	
☐	☒	The exact sample size (*n*) for each experimental group/condition, given as a discrete number and unit of measurement
☐	☒	A statement on whether measurements were taken from distinct samples or whether the same sample was measured repeatedly
☐	☒	The statistical test(s) used AND whether they are one- or two-sided<br>*Only common tests should be described solely by name; describe more complex techniques in the Methods section.*
☐	☒	A description of all covariates tested
☐	☒	A description of any assumptions or corrections, such as tests of normality and adjustment for multiple comparisons
☐	☒	A full description of the statistical parameters including central tendency (e.g. means) or other basic estimates (e.g. regression coefficient) AND variation (e.g. standard deviation) or associated estimates of uncertainty (e.g. confidence intervals)
☐	☒	For null hypothesis testing, the test statistic (e.g. *F*, *t*, *r*) with confidence intervals, effect sizes, degrees of freedom and *P* value noted<br>*Give P values as exact values whenever suitable.*
☒	☐	For Bayesian analysis, information on the choice of priors and Markov chain Monte Carlo settings
☒	☐	For hierarchical and complex designs, identification of the appropriate level for tests and full reporting of outcomes
☒	☐	Estimates of effect sizes (e.g. Cohen's *d*, Pearson's *r*), indicating how they were calculated

Our web collection on statistics for biologists contains articles on many of the points above.

Software and code

Policy information about availability of computer code

Data collection	No open source computer code was used for data collection
Data analysis	SAS version 9.4 was used for data analyses in this study

For manuscripts utilizing custom algorithms or software that are central to the research but not yet described in published literature, software must be made available to editors and reviewers. We strongly encourage code deposition in a community repository (e.g. GitHub). See the Nature Research guidelines for submitting code & software for further information.

Data

Policy information about availability of data

All manuscripts must include a data availability statement. This statement should provide the following information, where applicable:

- Accession codes, unique identifiers, or web links for publicly available datasets
- A list of figures that have associated raw data
- A description of any restrictions on data availability

Within 6 months of this publication, anonymized individual participant data, the annotated case report form, protocol, reporting and analysis plan, data set specifications, raw dataset, analysis-ready dataset, and clinical study report will be available for research proposals approved by an independent review committee. Proposals should be submitted to www.clinicalstudydatarequest.com. A data access agreement will be required.

Field-specific reporting

Please select the one below that is the best fit for your research. If you are not sure, read the appropriate sections before making your selection.

☒ Life sciences ☐ Behavioural & social sciences ☐ Ecological, evolutionary & environmental sciences

For a reference copy of the document with all sections, see nature.com/documents/nr-reporting-summary-flat.pdf

Life sciences study design

All studies must disclose on these points even when the disclosure is negative.

Sample size	There is no statistical rationale for the selected sample size of eight participants per cohort. The sample size was based on prior experience with other members of the drug class to ensure an adequate initial assessment of the safety and tolerability of while minimizing the number of patients unnecessarily exposed to the drug. The protocol included the ability to repeat a cohort if additional data at a specified cohort was warranted.
Data exclusions	Figure 2: one patient in the GSK3228836 mg arm discontinued on study Day 8 and is not shown. This patient discontinued treatment and also withdrew from the study; as such they were not assessed beyond Day 8 and are not shown in this figure (exclusion not prespecified). This patient was also excluded from the per protocol population (according to prespecified criteria.
Replication	Clinical trial data were derived from a total of 31 patients, and data from all 31 patients who were included in all analyses presented in this manuscript (except for 1 patient described in "Data exclusions" above). It was not feasible to replicate the data as this would require repeating the clinical trial.
Randomization	Patients were randomized (3:1 within each dose cohort) to GSK3228836 or placebo according to the randomization schedule (permuted block). The investigator (or designee) obtained the unique study treatment number via an interactive voice/internet response system.
Blinding	All participants, study monitors, study center personnel, and contract research organization personnel were blinded to treatment assignment.

Reporting for specific materials, systems and methods

We require information from authors about some types of materials, experimental systems and methods used in many studies. Here, indicate whether each material, system or method listed is relevant to your study. If you are not sure if a list item applies to your research, read the appropriate section before selecting a response.

Materials & experimental systems

n/a	Involved in the study	
☐	☒	Antibodies
☒	☐	Eukaryotic cell lines
☒	☐	Palaeontology and archaeology
☒	☐	Animals and other organisms
☐	☒	Human research participants
☐	☒	Clinical data
☒	☐	Dual use research of concern

Methods

n/a	Involved in the study	
☒	☐	ChIP-seq
☒	☐	Flow cytometry
☒	☐	MRI-based neuroimaging

Antibodies

Antibodies used	None
Validation	None

Human research participants

Policy information about studies involving human research participants

Population characteristics	Adult patients with chronic hepatitis B virus (HBV) infection who fulfilled the following inclusion criteria were eligible to participate in the study: Chronic HBV infection ≥6 months and serum hepatitis B surface antigen (HBsAg) ≥50 IU/mL; both hepatitis e antigen (HBeAg) positive and negative patients could participate. Treatment-naïve patients: Plasma HBV DNA ≥2x103 IU/mL. On-nucleo(s)tide (On-NA) patients: HBV DNA adequately suppressed (plasma or serum HBV DNA below lower limit of quantification [20 IU/mL]) and currently taking stable tenofovir disoproxil fumarate or entecavir for ≥12 months and expected to continue taking without change through to the end of their participation in this study.<br><br>Patients meeting the following criteria were excluded: History of liver cirrhosis and/or evidence of cirrhosis, liver failure, liver

disease other than hepatitis B, Gilbert's syndrome or history of laboratory results consistent with Gilbert's syndrome, extrahepatic disorders possibly related to HBV immune complexes, excess alcohol consumption; co-infection with hepatitis C virus, hepatitis D virus or HIV; screening laboratory values of alanine aminotransferase and aspartate aminotransferase >5 x upper limit of normal. Treatment-naïve patients: current or prior receipt of anti-HBV NA therapy. Patients who have failed prior interferon treatment >6 months prior to screening may be evaluated for possible participation in the study. The full study inclusion and exclusion criteria are detailed in the manuscript's supplementary section.

Thirty-one patients with CHB who were either treatment-naïve (Cohorts 1–3, n=24) or receiving stable NA therapy (Cohort 4, on-NA patients; n=7) were enrolled in the study. Patient demographics and baseline characteristics were similar between treatment arms. Demographics and baseline clinical characteristics are shown in Table 1 of the manuscript.

Recruitment

Patients were enrolled from one center in Hong Kong and five centers in the Republic of Korea. The first patient was enrolled February 22, 2017; the last patient was enrolled on the 30 April, 2019; the last patient visit was December 18, 2019; and the study was completed on December 19, 2019.

Ethics oversight

Details of the independent ethics committees/institutional review boards that approved the study are as follows:

Institutional Review Board of the University of Hong Kong/Hospital Authority Hong Kong West Cluster, Queen Mary Hospital 102 Pokfulam Road, Hong Kong;

Seoul National University Hospital Institutional Review Board, 101 Daehak-ro, Jongno-gu, Seoul 03080, Republic of Korea;

Kyungpook National Univeristy Institutional Review Board, 130 Dongdeok-ro, Jung-gu, Daegu 41944, Republic of Korea;

The Catholic University of Korea, Seoul St. Mary's Hospital Institutional Review Board 222, Banpo-daero, Seocho-gu, Seoul 06591, Republic of Korea;

Pusan National University Hospital Institutional Review Board, Department of Internal Medicine, 179 Gudeok-ro, Seo-gu, Busan 49241, Republic of Korea;

Korea University Ansan Hospital Institutional Review Board, 123 Jeokgeum-ro, Danwon-gu, Ansan-Si, Gyeonggi-do 15355 Republic of Korea;

Inje University Busan Paik Hospital Institutional Review Board, Bokji-ro 75, Busanjin-gu, Busan 47392, Republic of Korea

Note that full information on the approval of the study protocol must also be provided in the manuscript.

Clinical data

Policy information about clinical studies

All manuscripts should comply with the ICMJE guidelines for publication of clinical research and a completed CONSORT checklist must be included with all submissions.

Clinical trial registration

NCT02981602

Study protocol

The full study protocol can be accessed here: https://www.gsk-studyregister.com/en/trial-details/?id=205695

Data collection

Patients were enrolled from one study center in Hong Kong and five in the Republic of Korea. The first patient was enrolled February 22, 2017; last patient visit was December 18, 2019.

Six doses of GSK3228836 or placebo were administered via subcutaneous injection during the 4-week treatment period on study Days 1, 4, 8, 11, 15, and 22; patients were followed for 26 weeks until Day 211. On Day 29, 7 days after the last dose of study drug (GSK3328836 or placebo), the effects of treatment were assessed. The endpoint assessment for antiviral activity was on Day 29. The final study visit was Day 211. The 'End-of-Study' was defined as the last patient, last visit (Dec 18, 2019).

Blood samples for quantitative HBsAg, HBeAg, and HBV DNA measurement were collected at screening, pre-dose on Days 1, 15, and 29, and anytime on Days 23, 36, 57, 85, 113, and 211. Blood samples for categorical anti-HBs and anti-HBe antibodies measurement were collected at screening, pre-dose on Days 1 and 29, and anytime on Days 57, 113, and 211.

Outcomes

The primary objective was to examine the safety and tolerability of GSK3228836 administration in treatment-naïve participants with chronic hepatitis B infection. This was assessed via incidence of adverse events [AEs], and findings from clinical laboratory tests, vital signs and body weight, physical examination, electrocardiogram, and concomitant medication usage.

Secondary objectives were:
- Examine the effects of GSK3228836 administration on plasma HBV DNA concentration (assessed by change from baseline to Day 29 and Week 31)
- Examine the effects of GSK3228836 administration on serum HBsAg concentration (assessed by change from baseline to Day 29 and Week 31, and the proportion of participants with HBsAg loss at Day 29 and at Week 31)
- Examine the effect of GSK3228836 administration on serum HBeAg concentration in patients who were HBeAg-positive at baseline (assessed by change from baseline to Day 29 and Week 31, proportion of participants with HBeAg loss at Day 29 and at Week 31)
- Assess plasma pharmacokinetics of GSK3228836 in patients with chronic HBV infection (assessed by plasma concentrations of GSK3228836 using validated bioanalytical methods)
- Describe the safety and tolerability of tenofovir disoproxil fumarate (and entecavir, if administered) therapy following conclusion of GSK3228836 administration (assessed by the incidence of adverse events after Day 29)

Exploratory objective:
- Describe the rate of seroconversion to anti-HBs or anti-HBe antibody-positive during treatment with GSK3228836 and then during subsequent treatment with TDF (or ETV if administered) (assessed by the proportion of patients with antibody positivity at Day 29 and at Week 31)

This checklist template is licensed under a Creative Commons Attribution 4.0 International License, which permits use, sharing, adaptation, distribution and reproduction in any medium or format, as long as you give appropriate credit to the original author(s) and the source, provide a link to the Creative Commons license, and indicate if changes were made. The images or other third party material in this article are included in the article's Creative Commons license, unless indicated otherwise in a credit line to the material. If material is not included in the article's Creative Commons license and your intended use is not permitted by statutory regulation or exceeds the permitted use, you will need to obtain permission directly from the copyright holder. To view a copy of this license, visit http://creativecommons.org/licenses/by/4.0/
